# StrainMake: reproducible hybrid metagenomics with MAG recovery and strain-level resolution

**DOI:** 10.1093/bioinformatics/btag212

**Published:** 2026-05-07

**Authors:** Baptiste Hennecart, Eugeni Belda, Raynald de Lahondès, Jean-Daniel Zucker, Edi Prifti

**Affiliations:** IRD, Sorbonne Université, UMMISCO, Paris, 75005, France; GMT Science, Paris, 75013, France; IRD, Sorbonne Université, UMMISCO, Paris, 75005, France; Sorbonne University, INSERM, nutrition and obesities: systemic approaches, NutriOmique, Paris, 75013, France; GMT Science, Paris, 75013, France; IRD, Sorbonne Université, UMMISCO, Paris, 75005, France; Sorbonne University, INSERM, nutrition and obesities: systemic approaches, NutriOmique, Paris, 75013, France; IRD, Sorbonne Université, UMMISCO, Paris, 75005, France; Sorbonne University, INSERM, nutrition and obesities: systemic approaches, NutriOmique, Paris, 75013, France

## Abstract

**Summary:**

Metagenomic workflows involve complex multi-step analyses, from quality control and assembly to binning, annotation, and strain-level profiling. Few existing metagenomic pipelines achieve the combination of flexibility, reproducibility, and hybrid assembly support within a unified workflow. We present StrainMake, a Snakemake-based workflow for de novo metagenomic analysis from short, long, or hybrid sequencing data. StrainMake integrates widely used tools across all major steps—quality control, assembly, binning, dereplication, taxonomic and functional annotation—while also providing non-redundant gene catalogues, community-scale metabolic models, and strain-level microdiversity metrics. The modular design enables the use of alternative tools, scalable execution on HPC systems, and full reproducibility through Snakemake and Conda.

**Results:**

Applied to the CAMI II strain-madness dataset, StrainMake produced high-quality assemblies and metagenome-assembled genomes (MAGs), while enabling strain-resolved comparisons across samples. Hybrid assemblies improved contiguity, whereas short-read assemblies offered faster runtimes, illustrating the workflow’s benchmarking capacity.

**Availability and implementation:**

StrainMake is open source and available at https://github.com/UMMISCO/strainmake, together with comprehensive documentation. Generated data are deposited in Zenodo (doi: 10.5281/zenodo.16950162).

## 1 Introduction

Recent advances in metagenomics have shifted the focus from species- to strain-level resolution. While early microbial community studies relied on marker-gene surveys or species-level metagenome-assembled genomes (MAGs), it is now clear that strain heterogeneity strongly impacts ecological functions, host interactions, and clinical outcomes (Olm et al. 2021, Kim et al. 2024). For example, closely related strains may differ in antibiotic resistance, pathogenicity, and metabolic capabilities, making strain-aware analyses essential for both environmental and biomedical research.

The concept of “strain” in metagenomic studies is not uniquely defined and depends on the analytical scale considered. Strain-level variation may refer to different concepts such as genome-wide divergence between closely related genomes (Van Rossum et al. 2020), fine-scale sequence variation within populations (Olm et al. 2021), or the presence of distinct haplotypes representing coexisting genetic lineages (Shaw et al. 2024). These complementary perspectives reflect the continuum of within-species diversity observed in natural and host-associated microbiomes.

Achieving strain-level resolution remains challenging. It requires not only accurate assembly and binning, but also sensitive methods, capable of capturing within-species microdiversity, including single-nucleotide variants, haplotypes, or gene-content differences (Yang et al. 2021, Sanchez et al. 2024). The integration of short- and long-read sequencing has further accelerated this shift, as hybrid assemblies enhance contiguity and genome recovery, enabling the reconstruction of multiple coexisting strains (Goussarov et al. 2024, Kang et al. 2024). However, such analyses require complex workflows that orchestrate numerous tools and remain difficult to reproduce.

Existing pipelines partially address this challenge. Frameworks such as ATLAS (Kieser et al. 2020) and MetaWRAP (Uritskiy et al. 2018) provide end-to-end workflows for species-level MAG recovery but are largely limited to short reads. SqueezeMeta (Tamames and Puente-Sánchez 2018) offers an automated metagenomic analysis framework, yet it remains focused on species-level reconstruction and lacks support for strain-level analyses. Aviary (Newell et al. 2024) extends to hybrid assemblies and some strain-aware analyses, yet it lacks modules for building non-redundant gene catalogues or genome-scale metabolic models. As a result, researchers often rely on ad hoc scripts to meet specific analytical needs, which hinders reproducibility, comparability, and scalability across studies.

Here, we present StrainMake, a workflow designed to make strain-level metagenomic analyses both accessible and reproducible. In this context, StrainMake does not enforce a single operational definition of a strain. Instead, it supports complementary strain-level analyses: genome-wide similarity, population microdiversity, and haplotype-level structure, allowing users to select the level of resolution most appropriate to their biological question and sequencing data. StrainMake integrates widely used tools for quality control, assembly, binning and refinement, dereplication, and annotation, while also providing modules for non-redundant gene catalogue reconstruction, strain-level microdiversity analysis, and metabolic modelling at both genome and community scales. It supports short-, long-, and hybrid-read sequencing data, executes alternative tools in parallel for benchmarking, and ensures reproducibility through Conda-managed environments. A comparative overview of StrainMake and existing pipelines cited above, highlighting key features and strain-level capabilities, is provided in Table 1, available as supplementary data at *Bioinformatics* online.

By emphasizing strain resolution and reproducibility, StrainMake fills a critical gap in existing frameworks. It offers a standardized yet flexible resource for applications ranging from microbial ecology to clinical microbiome research, where capturing strain-level diversity is increasingly recognized as essential.

## 2 Description

**Figure 1:**
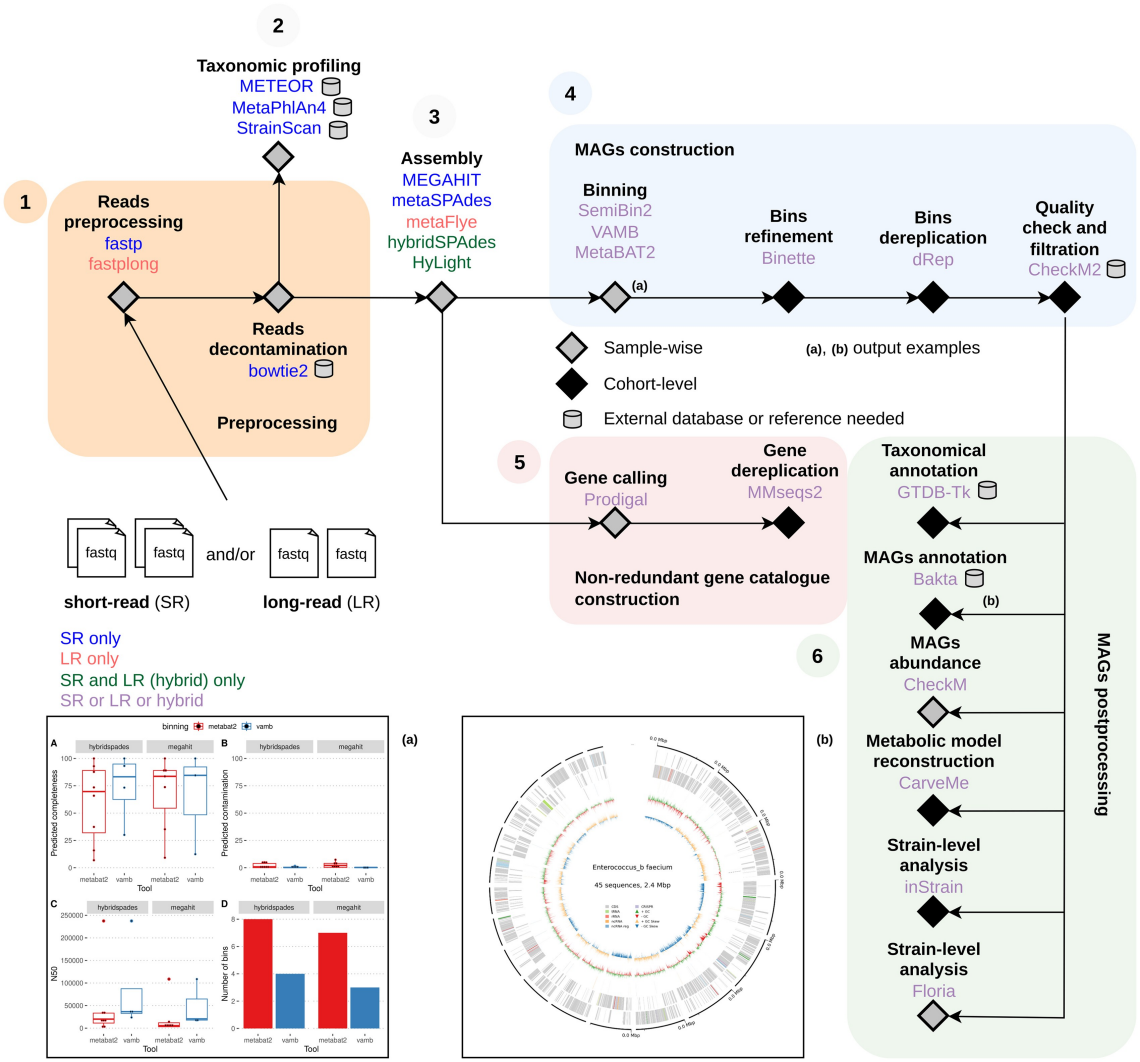
Overview of the StrainMake workflow. It accepts short-, long-, or hybrid-read sequencing data and comprises six main modules: (1) preprocessing, (2) direct taxonomic profiling, (3) assembly, (4) MAG construction, (5) non-redundant gene catalogue generation and (6) MAG postprocessing. Example outputs include: (a) comparison of binning statistics before refinement (by assembler and binner), and (b) functional annotation of a recovered MAG using Bakta.

StrainMake is a modular workflow for de novo metagenomics, implemented in Snakemake (Mölder et al. 2021) with Conda-based environments to ensure reproducibility. It supports short-, long- (Oxford Nanopore or PacBio), and hybrid-read sequencing data, and integrates state-of-the-art tools for all major steps: quality control, assembly, gene prediction, binning and refinement, dereplication, annotation, strain-level analysis, and taxonomic profiling (Fig. 1). Users can flexibly select among different tools, and alternative configurations can be executed in parallel, making the workflow suitable for benchmarking and scalable deployment on HPC systems.

### 2.1 Major steps

StrainMake is structured into modular components that span the entire strain-level metagenomic analysis process. Each step can be customized or executed independently, providing flexibility while maintaining reproducibility. The main modules are detailed below (Fig. 1).

**Preprocessing and assembly:** Raw reads are filtered and quality-checked before assembly. Multiple short-, long-, and hybrid-read assemblers are supported and can be run in parallel. Assembly statistics are computed automatically, and contigs below user-defined thresholds are removed.
**Gene catalogues:** Genes are predicted from contigs, pooled across samples, and dereplicated into a non-redundant catalogue. This provides a shared reference for downstream functional annotation and abundance profiling.
**Binning and MAG recovery:** Contigs are binned with alternative algorithms and refined to maximize quality. Bins are then dereplicated across samples to yield high-quality MAGs. By default, dereplication is performed at both 95% ANI (species level) and 97% ANI (strain level). MAGs are subsequently taxonomically classified and annotated.
**Abundance and metabolic models:** Relative abundances of MAGs are estimated from read coverage. Genome-scale metabolic models are reconstructed for both individual MAGs and merged communities, linking genome recovery to functional potential.
**Strain-level analysis:** Population microdiversity is characterized through single-nucleotide variants, nucleotide diversity, and haplotypes. Comparisons across samples allow strain tracking and the detection of shared populations, complementing MAG-based reconstruction.
**Taxonomic profiling and reporting:** Direct taxonomic profiling from reads is supported for rapid community overviews. Optionally, users may generate a MultiQC (Ewels et al. 2016) report summarizing outputs from all supported tools, enhancing transparency and reproducibility.

### 2.2 Implementation

The pipeline is fully configurable through a YAML file, where users specify tool choices, reference databases, and thresholds. It runs seamlessly on local machines, HPC clusters, or cloud environments, with automatic dependency management. Outputs are organized in a standardized structure, making them easy to share and reuse. Detailed software versions and parameter defaults are provided in the Supplementary Information.

## 3 Application

StrainMake was evaluated on ten simulated samples from the CAMI II “strain-madness” dataset (Fritz et al. 2021), each containing both Illumina short reads and PacBio long reads. We ran the full workflow with MEGAHIT for short-read assemblies, hybridSPAdes for hybrid assemblies, and both VAMB and MetaBAT2 for binning. In this setup, StrainMake executes tasks in parallel across assemblers and binning tools, enabling straightforward benchmarking of alternative strategies.

Assemblies obtained with MEGAHIT were generally longer, whereas hybridSPAdes consistently produced more contiguous assemblies, with the largest contigs and higher N50 values (Castro and Ng 2017) (Fig. 4, available as supplementary data at *Bioinformatics* online). As expected, hybrid assemblies required more computation time, while MEGAHIT ran faster but produced shorter contigs (Figs. 4B and 9A, available as supplementary data at *Bioinformatics* online). This comparison illustrates how StrainMake enables benchmarking of the trade-off between assembly contiguity and runtime across assemblers.

For binning, MetaBAT2 was faster and required fewer computational resources, while VAMB often produced bins of higher quality (Figs. 5 and 9, available as supplementary data at *Bioinformatics* online). After refinement and dereplication, we obtained a catalogue of high-quality MAGs (Fig. 6, available as supplementary data at *Bioinformatics* online), which were subsequently annotated taxonomically and functionally (Fig. 7, available as supplementary data at *Bioinformatics* online).

Recovered MAGs served as references for strain-level analyses with inStrain and Floria. For instance, StrainMake enabled comparisons of within-species diversity across assemblers, as illustrated by popANI distributions for *Enterococcus B faecium* (Fig. 8B, available as supplementary data at *Bioinformatics* online) and the number of strain clusters reconstructed per species (Fig. 8C, available as supplementary data at *Bioinformatics* online). These results highlight the ability of StrainMake to achieve genome-level recovery and capture fine-scale strain heterogeneity within microbial communities.

To assess the robustness of these results, we further evaluated StrainMake on a real human gut metagenomic cohort (90 samples, PRJNA961076) at three sequencing depths, and performed dedicated experiments on sequencing coverage and cross-sample contamination (Supplementary Information). Strain-level analyses remained reliable at ≥ 10 × genome coverage and ≤ 5% cross-contamination, while lower coverage or higher contamination primarily affected strain-count estimates for low-prevalence taxa. These findings provide practical guidance on the conditions under which StrainMake’s strain-level outputs can be interpreted with confidence.

## 4 Conclusion

We developed StrainMake, a reproducible and flexible workflow for de novo metagenomic analysis that accommodates short-, long-, and hybrid-read sequencing data. By enabling the parallel execution of alternative tools, it facilitates systematic benchmarking and empowers users to tailor analyses to their datasets. Beyond high-quality MAG recovery, StrainMake extends to strain-level investigations, non-redundant gene catalogue reconstruction, and both genome- and community-scale metabolic modelling, thereby linking taxonomic resolution with functional insight. Its modular design, transparent rule execution, and Conda-managed environments ensure reproducibility across computing platforms, from local machines to HPC clusters. Together, these features position StrainMake as a comprehensive and community-ready resource for genome- and strain-resolved metagenomics, addressing the growing need for reproducible, strain-aware, and hybrid-capable workflows in microbial ecology and clinical microbiome research.

## Supplementary Material

btag212_Supplementary_Data

## Data Availability

StrainMake, together with documentation and tutorials, is openly available at https://github.com/UMMISCO/strainmake. The source code is also archived in Zenodo under DOI 10.5281/zenodo.18196707. All assemblies, quality metrics, MAGs, annotations, and strain-level results reported in this article have been deposited in Zenodo under DOI 10.5281/zenodo.16950162.
