# Supplementary material for: StrainMake: reproducible hybrid metagenomics with MAG recovery and strain-level resolution
Source: Bioinformatics. 2026 May 7;42(5):btag212. doi: 10.1093/bioinformatics/btag212 (PMC13188985; doi:10.1093/bioinformatics/btag212)
Supplement: btag212_Supplementary_Data [file btag212_supplementary_data.pdf]

Supplementary Information for:

StrainMake: reproducible hybrid metagenomics with MAG recovery and strain-level resolution

Baptiste Hennecart, Eugeni Belda, Raynald de Lahondès,
Jean-Daniel Zucker, Edi Prifti

Contents

1 Tool list and versions **4**

2 Metagenomic pipelines feature comparison **4**

3 Extended methods of StrainMake **6**
- 3.1 Preprocessing . . . 6
- 3.2 Assembly . . . 6
- 3.3 Gene catalogue . . . 6
- 3.4 Binning and bin refinement . . . 6
- 3.5 Bin postprocessing . . . 7
- 3.6 MAG abundance . . . 7
- 3.7 Metabolic models . . . 7
- 3.8 Strain-level analysis . . . 7
- 3.9 Taxonomic profiling . . . 7

4 Guidelines for parameter selection **7**

5 Benchmarking results **10**
- 5.1 Technical details . . . 10
- 5.2 CAMI II strain-madness benchmark . . . 10

6 Analysis of CRC cohort PRJNA961076 **16**

7 Impact of sequencing coverage **20**

8 Impact of cross-contamination **22**

List of Figures

- S1 Impact of contig-length filtering on assembly metrics . . . 9
- S2 Impact of contig-length filtering on gene catalogue size . . . 9
- S3 Impact of contig-length filtering on recovered MAGs . . . 10
- S4 Benchmarking of metagenomic assemblers (CAMI II) . . . 11
- S5 Benchmarking of metagenomic binners prior to refinement (CAMI II) . . . 12
- S6 Refined bins and MAG quality across assemblers (CAMI II) . . . 13
- S7 MAG annotation comparison across assembly strategies (CAMI II) . . . 14
- S8 Gene catalogue construction and strain-level analyses (CAMI II) . . . 15

S9 Computational performance benchmarking (CAMI II) 16
S10 Sequencing depth of the PRJNA961076 cohort 17
S11 Assembly metrics for the PRJNA961076 cohort 18
S12 MAG-level summaries for the PRJNA961076 cohort 19
S13 Gene catalogue and strain-level similarity for the PRJNA961076 cohort . . 20
S14 Species recovery across genome-coverage conditions 21
S15 Effect of sequencing coverage on Floria strain-count estimation 22
S16 Effect of contamination on assembly metrics and bin recovery 24
S17 MAG quality metrics across contamination levels 24
S18 Effect of contamination on strain-level estimates and microdiversity 25
S19 Pairwise Wilcoxon tests of strain counts across contamination scenarios . . 26

List of Tables

S1 Feature comparison of StrainMake and existing metagenomic workflows . . 5
S2 Bacterial genomes used in the coverage simulation 21

List of abbreviations

Abbreviation	Definition
ANI	Average Nucleotide Identity
BH	Benjamini–Hochberg (correction for multiple testing)
bp	Base pair(s)
CAMI	Critical Assessment of Metagenome Interpretation
CC	Cloud Computing
CLI	Command-Line Interface
CRC	Colorectal Cancer
FDR	False Discovery Rate
GTDB	Genome Taxonomy Database
HPC	High-Performance Computing
MAG	Metagenome-Assembled Genome
PacBio	Pacific Biosciences
popANI	Population Average Nucleotide Identity
SNV	Single-Nucleotide Variant
UHGG	Unified Human Gastrointestinal Genome

1 Tool list and versions

- **Quality control:** FastQC v0.12.0, fastp v0.23.4, fastplong v0.3.0, Bowtie2 v2.5.4.
- **Assembly:** MEGAHIT v1.2.9, metaSPAdes v3.15.5, metaFlye v2.9.6, hybridSPAdes v3.15.5, HyLight v1.0.0.
- **Assembly quality check:** MetaQUAST v5.2.0.
- **Gene catalogue:** prodigal v2.6.3, MMseqs2 v15.6f452.
- **Binning:** MetaBAT2 v2.17, VAMB v4.1.3, SemiBin2 v2.1.0.
- **Refinement:** Binette v1.1.2.
- **Dereplication:** dRep v3.5.0, CheckM2 v1.0.2.
- **Annotation:** GTDB-Tk v2.4.1, Bakta v1.11.3.
- **Abundance:** CheckM v1.2.2.
- **Strain analysis:** inStrain v1.9.1, Floria v0.0.2, minimap2 v2.28.
- **Metabolic models:** CarveMe v1.6.2.
- **Profiling:** MetaPhlAn v4.2.2, METEOR v2.0.21.

2 Metagenomic pipelines feature comparison

Feature	StrainMake	MetaWRAP	Aviary	SqueezeMeta
**Short reads**	YES	YES	YES	YES
**Long reads**	YES	NO	YES	YES
**Hybrid assembly**	YES	NO	YES	YES
**Multi-sample**	YES	YES	YES	YES
**Assembly / binning strategy**	Single	Single/Co	Single	Single/Co
**Bin refinement**	YES	YES	YES	YES
**Strain-level (profiling)**	YES	NO	NO	NO
**Strain-level (variant-based, microdiversity)**	YES	NO	YES	YES
**Community metabolic models**	YES	NO	NO	NO
**Computational resources**	Local/HPC/CC	Local/HPC	Local/HPC/CC	Local/HPC
**Interface**	CLI	CLI	CLI	CLI
**Workflow manager**	Snakemake	–	Snakemake	–
**Software execution**	Conda	Conda/Docker	Conda	Conda

Table S1: Feature comparison of StrainMake and existing metagenomic workflows. Information for MetaWRAP [Uritskiy et al., 2018], Aviary [Newell et al., 2024], and SqueezeMeta [Tamames and Puente-Sánchez, 2019] was informed by the review of Yepes-García and Falquet [2026].

3 Extended methods of StrainMake

3.1 Preprocessing

Preprocessing of raw short reads, such as filtering of low-quality reads and adapter trimming, is done using fastp v0.23.4 [Chen, 2023]. The following parameters are used: `--detect_adapter_for_pe --length_required 50 --qualified_quality_phred 15 --compression 2`. For the decontamination step, short reads are mapped to a reference genome using Bowtie2 v2.5.4 [Langmead and Salzberg, 2012] with default parameters. Reads that do not map to the reference genome are retained for downstream analysis. By default, the CHM13 v2 human reference genome is used for decontamination. FastQC reports before and after preprocessing are generated for short reads using FastQC v0.12.0 [Andrews, 2025].

Long reads are preprocessed using fastplong v0.3.0 [Chen, 2023]. The following parameters are used: `--length_required 1000 --qualified_quality_phred 12 --compression 2`.

3.2 Assembly

Short-read assembly can be carried out using either MEGAHIT v1.2.9 [Li et al., 2015] (default parameters, adjustable by the user) or metaSPAdes v3.15.5 [Nurk et al., 2017], invoked via `spades.py` with the `--meta` flag (default parameters, with 50 GB of memory, adjustable by the user). Long-read assembly can be carried out using metaFlye v2.9.6 [Kolmogorov et al., 2020], invoked via `flye` with the `--meta` flag (default parameters, adjustable by the user). Hybrid assembly can be carried out using hybridSPAdes v3.15.5 [Antipov et al., 2016] (default parameters, with 50 GB of memory, adjustable by the user) or HyLight v1.0.0 [Kang et al., 2024] (default parameters, adjustable by the user).

When multiple assemblers are specified, assembly processes and downstream tasks are executed in parallel. By default, contigs shorter than 500 bp are excluded from the final assembly using seqkit v2.8 [Shen et al., 2024] when the assembler does not enforce a minimum contig length.

MetaQUAST v5.2.0 [Mikheenko et al., 2016] is used with the parameter `--max-ref-number 0` to compute assembly quality metrics and statistics, such as N50, L50, auN, and largest contig size.

3.3 Gene catalogue

StrainMake performs gene calling on assemblies using prodigal v2.6.3 [Hyatt et al., 2010] with the `-p meta` option, on a per-sample basis. Genes are then pooled into a single FASTA file using seqkit and dereplicated with MMseqs2 v15.6f452's `easy-cluster` subcommand [Steinegger and Söding, 2017]. By default, the following MMseqs2 parameters are used: minimum sequence identity of 0.95 and minimum fraction of aligned bases of 0.90, along with the options `--alignment-mode 3 --cov-mode 1 --dbtype 2`. By default, genes shorter than 100 bp are discarded. This yields a non-redundant gene catalogue for the cohort.

3.4 Binning and bin refinement

Contig binning is performed using VAMB v4.1.3 [Nissen et al., 2021] (by default with a minimum bin size of 200,000 bp, 50 epochs, and a start batch size of 10), SemiBin2 v2.1.0 [Pan et al., 2023], and/or MetaBAT2 v2.17 [Kang et al., 2019] (by default with a minimum bin size of 200,000 bp, minimum contig size of 1,500 bp, and minimum mean coverage of 1). The quality of the resulting bins is evaluated using CheckM2 v1.0.2 [Chklovski et al., 2023], which estimates genome completeness and contamination using machine learning.

When multiple binning tools are used, bins are subsequently pooled by sample and refined using Binette v1.1.2 [Mainguy and Hoede, 2024] to produce a higher-quality bin set for each sample.

3.5 Bin postprocessing

To obtain final metagenome-assembled genomes (MAGs), bins across all samples are pooled and dereplicated using dRep v3.5.0 [Olm et al., 2017] with ANImf as the default comparison algorithm. By default, two sets of MAGs are produced: one at a 95% ANI threshold (species level) and one at 97% (strain level). Their contamination and completeness are assessed using CheckM2; by default, only those with completeness > 75% and contamination < 10% are retained.

Species-level MAGs are taxonomically annotated using GTDB-Tk v2.4.1 [Chaumeil et al., 2022], with the `--skip_ani_screen --pplacer_cpus 1` options. Bacterial MAGs are annotated using Bakta v1.11.3 [Schwengers et al., 2021].

3.6 MAG abundance

The abundance of species-level MAGs is estimated using CheckM v1.2.2 [Parks et al., 2015], through the `coverage` and `profile` modules.

3.7 Metabolic models

Metabolic model reconstruction is carried out on MAGs obtained at different ANI dereplication thresholds using CarveMe v1.6.2 [Machado et al., 2018]. Genome-scale models are built using the `carve` command, while community-level models are generated with the `merge_community` command using the resulting individual models.

3.8 Strain-level analysis

Strain-level microdiversity metrics, including nucleotide diversity and single-nucleotide variants (SNVs), are computed using inStrain v1.9.1 [Olm et al., 2021] with species-level MAGs as references by default. minimap2 v2.28 [Li, 2018] is used to map metagenomic reads to the references. Analyses are performed at multiple genomic scales—gene-level and genome-level—using the `profile` command on each sample. Strain-level comparisons across samples are then conducted using the `compare` command.

Strain-level phasing is performed by sample using Floria v0.0.2 [Shaw et al., 2024].

3.9 Taxonomic profiling

StrainMake also supports direct species-level taxonomic profiling using either METEOR v2.0.21 [Ghozlane et al., 2025] or MetaPhlAn v4.2.2 [Blanco-Míguez et al., 2023]. Strain-level profiling for a given species can also be performed via StrainMake using StrainScan v1.0.14 [Liao et al., 2023].

4 Guidelines for parameter selection

- **Preprocessing.** StrainMake provides conservative default settings (e.g., short-read minimum length of 50 bp and minimum Phred quality score of 15 in fastp). Increasing these thresholds can be useful for datasets with low-quality tails or short-read artifacts, at the cost of reduced sequencing depth. Additional platform-specific trimming can also be enabled (e.g., polyG tail trimming on specific Illumina instruments).

- **Assembly.** StrainMake performs post-assembly contig-length filtering to exclude contigs shorter than 500 bp by default (when the assembler does not enforce a minimum length). This removes very short, often low-information contigs, limits downstream computational burden, and facilitates fairer comparisons across assemblers and sequencing strategies. Setting the minimum accepted contig length to 1 bp effectively disables this filter.

  To assess the impact of this parameter, we conducted a contig-length filtering experiment on the same 10 CAMI II strain-madness metagenomes used in the benchmarking section (no filtering, 500 bp, 1,000 bp, 10,000 bp). Assembly metrics were significantly affected by the filtering threshold (Friedman tests: $p < 0.001$ for total assembly length, N50, and L50). Increasing the threshold also reduced gene calling output and gene catalogue size (all genes: 8,039,484 with no filtering vs 128,601 at 10,000 bp; non-redundant catalogue: 1,223,851 with no filtering vs 35,298 at 10,000 bp). In contrast, MAG quality distributions were stable across conditions (Kruskal–Wallis tests: completeness, contamination, and MAG N50 all $p > 0.05$), although the number of recovered MAGs varied. Overall, these results support the default 500 bp threshold as a pragmatic trade-off between retaining sequence content (and gene diversity) and reducing low-information contigs.

  StrainMake allows users to select between assemblers depending on their priorities. For short reads, SPAdes is recommended for genome-centric applications requiring long contigs, whereas MEGAHIT is preferred for large-scale or diversity-focused analyses, especially when computational resources are limited or when capturing community-level and strain-level variation is critical [Vollmers et al., 2017, van der Walt et al., 2017].

  Concerning assembler-specific settings, users are encouraged to consult the recommendations of each tool (e.g., selection of k-mer sizes for MEGAHIT).

- **Binning.** Recent benchmarking studies have highlighted that no single metagenomic binning tool consistently outperforms others across all data types and analytical settings [Han et al., 2025]. Instead, binning performance strongly depends on sequencing strategy (short-read, long-read, or hybrid) and assembly characteristics. In particular, hybrid assemblies and multi-sample binning strategies generally yield higher recovery of high-quality MAGs and better capture of species and strain diversity. Based on these observations, StrainMake integrates complementary binning approaches: MetaBAT2 (widely used and computationally efficient), VAMB (deep learning–based representation learning), and SemiBin2 (contrastive learning–based binning with strong performance across diverse datasets).

- **Dereplication.** Default ANI thresholds for bin dereplication were chosen following the literature. The 95% ANI threshold is commonly used as a species-level cutoff for whole-genome ANI analyses [Van Rossum et al., 2020], while 97% is often used as a conservative minimum threshold for strain-level comparisons.

- **MAG quality filtering.** Completeness and contamination thresholds should be determined by the intended downstream analyses. Common guidance classifies MAGs as high-quality ($\geq$ 90% completeness, $\leq$ 5% contamination), medium-quality ($\geq$ 50% completeness, $\leq$ 10% contamination), or low-quality [Cansdale and McInerney, 2024]. In StrainMake, the default filters ($\geq$ 75% completeness and $\leq$ 10% contamination) were chosen as a pragmatic compromise between genome completeness and contamination control. Users may adjust these thresholds to suit their objectives.

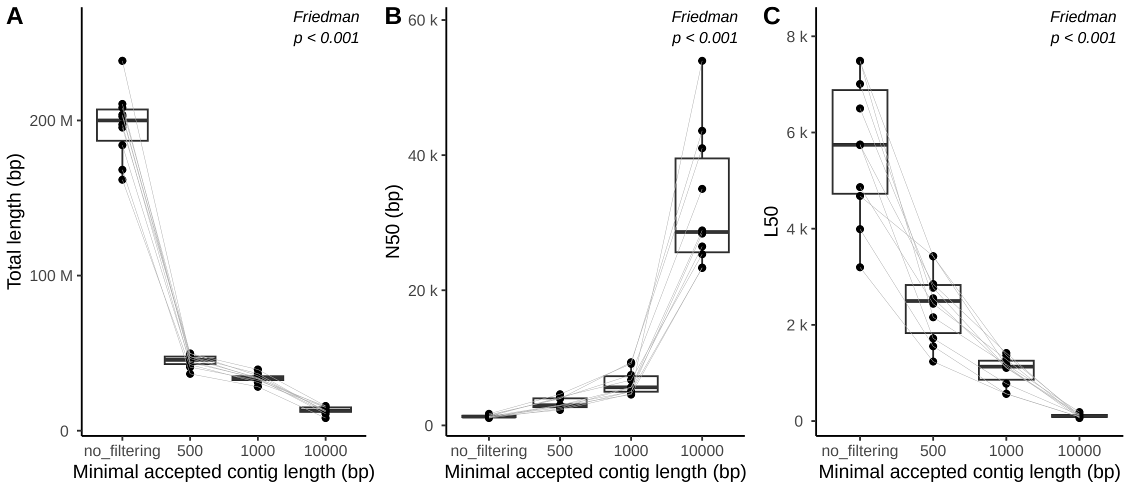

Figure S1: Impact of post-assembly contig-length filtering on assembly metrics. Assemblies were filtered by excluding contigs shorter than the indicated minimal accepted length (no filtering, 500 bp, 1,000 bp, 10,000 bp). **A.** Total assembly length (bp). **B.** N50 (bp). **C.** L50. Each point represents one assembly, and lines connect the same metagenome across filtering conditions. Friedman test for all three metrics: $p < 0.001$.

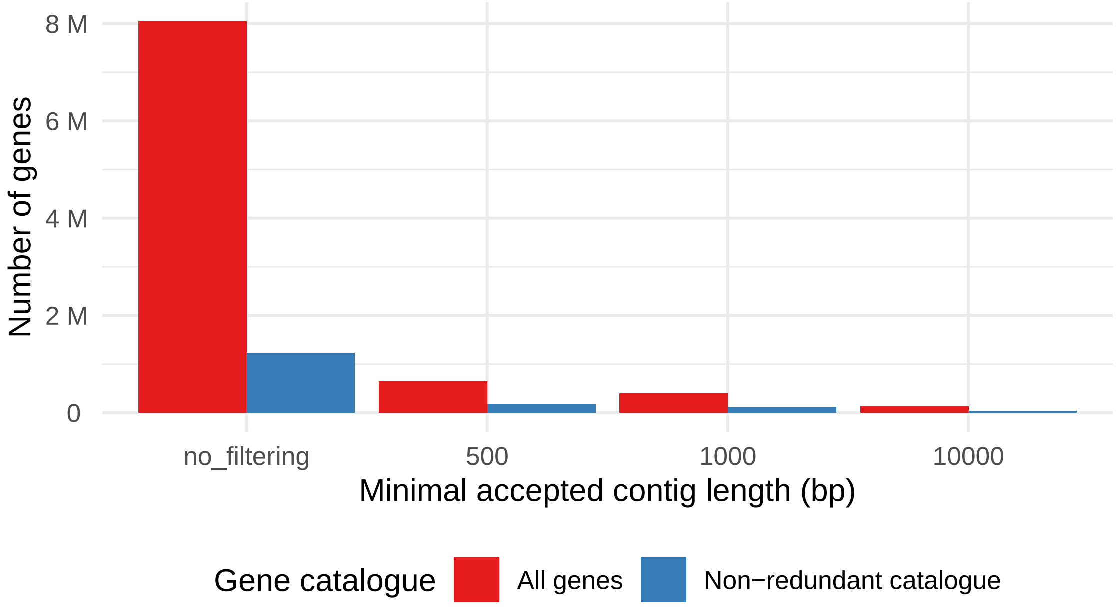

Figure S2: Impact of post-assembly contig-length filtering on gene catalogue size. Gene calling was performed on the filtered assemblies, and gene catalogues were constructed using StrainMake by pooling genes across samples ("All genes") and then clustering to generate a non-redundant catalogue ("Non-redundant catalogue").

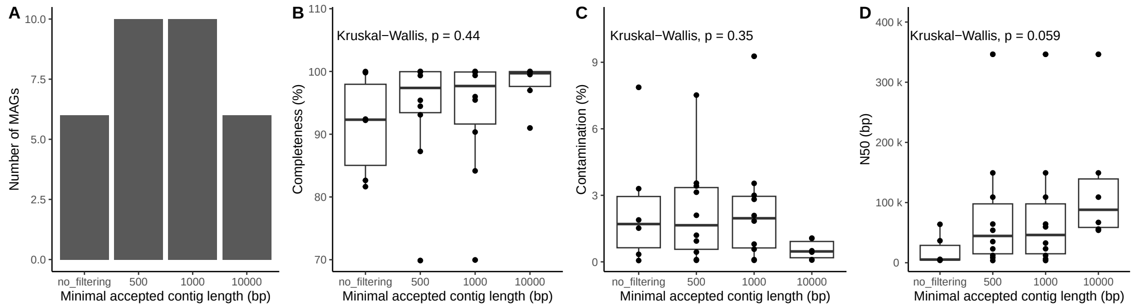

Figure S3: Impact of post-assembly contig-length filtering on recovered MAGs. MAGs were generated with the StrainMake pipeline from the filtered assemblies by binning with MetaBAT2 and VAMB, followed by refinement, pooling across samples, dereplication, and quality filtering. **A.** Number of MAGs obtained per filtering condition. **B.** CheckM2-predicted completeness (%). **C.** CheckM2-predicted contamination (%). **D.** MAG contig N50 (bp). Kruskal–Wallis tests: completeness, contamination, and N50 did not differ significantly across conditions (all $p > 0.05$).

5 Benchmarking results

5.1 Technical details

For the CAMI II benchmark, StrainMake was run on a server with the following specifications:

Specification	Value
OS	Linux 4.18
Architecture	x86_64
CPU	Intel Xeon E5-2680 v4 @ 2.40 GHz
Cores	56
RAM	251 GB

5.2 CAMI II strain-madness benchmark

StrainMake was evaluated on ten simulated samples from the CAMI II "strain-madness" dataset, each containing both Illumina short reads and PacBio long reads. We ran the full workflow using MEGAHIT for short-read assemblies and hybridSPAdes for hybrid assemblies, with VAMB and MetaBAT2 for contig binning.

Assemblies obtained with MEGAHIT generally had greater total length, whereas hybridSPAdes consistently produced more contiguous assemblies, with larger contigs and higher N50 values (Fig. S4). Hybrid assemblies required more computation time, while MEGAHIT ran faster but produced shorter contigs, illustrating the trade-off between contiguity and runtime (Fig. S9A–B).

For binning, MetaBAT2 was faster and required fewer computational resources, while VAMB often produced bins of higher quality prior to refinement (Fig. S5). After refinement with Binette and dereplication with dRep at 97% ANI, we obtained a catalogue of high-quality MAGs with high completeness and low contamination (Fig. S6). Recovered MAGs were subsequently annotated taxonomically with GTDB-Tk and functionally with Bakta. As an example, Bakta annotations of *Enterococcus B faecium* MAGs from short-read and hybrid assemblies are compared in Fig. S7, together with a whole-genome alignment against a reference genome.

Gene catalogues were constructed from each assembly strategy and compared (Fig. S8A). Species-level MAGs were then used as references for strain-level analyses with inStrain and Floria. The inStrain popANI distributions for *E. faecium* across samples revealed assembler-dependent variation in within-species diversity estimates (Fig. S8B). The number of strain-level clusters detected per species (Fig. S8C) and the deviation from the simulated number of strains (Fig. S8D) showed that strain diversity estimation remains challenging under very high strain complexity, but the workflow produces consistent and interpretable results. Overall, computational benchmarking confirmed that binning runtimes and CPU loads varied substantially across tools (Fig. S9C–D).

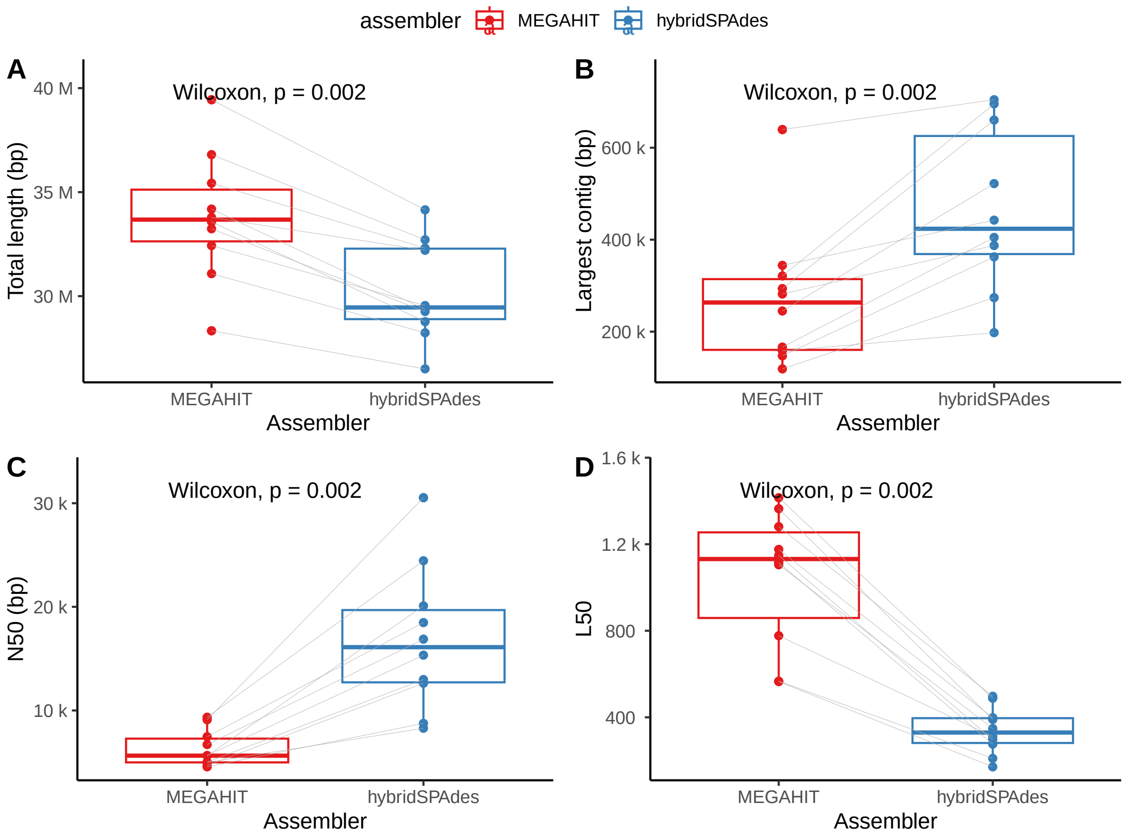

Figure S4: StrainMake facilitates straightforward benchmarking of metagenomic assemblers. **A.** Total assembly length (in base pairs). **B.** Largest contig length (in base pairs). **C.** N50 values (in base pairs). **D.** L50 values. Assemblies were generated either with MEGAHIT (short-read assemblies) or with hybridSPAdes (hybrid assemblies). Each point represents an individual assembly.

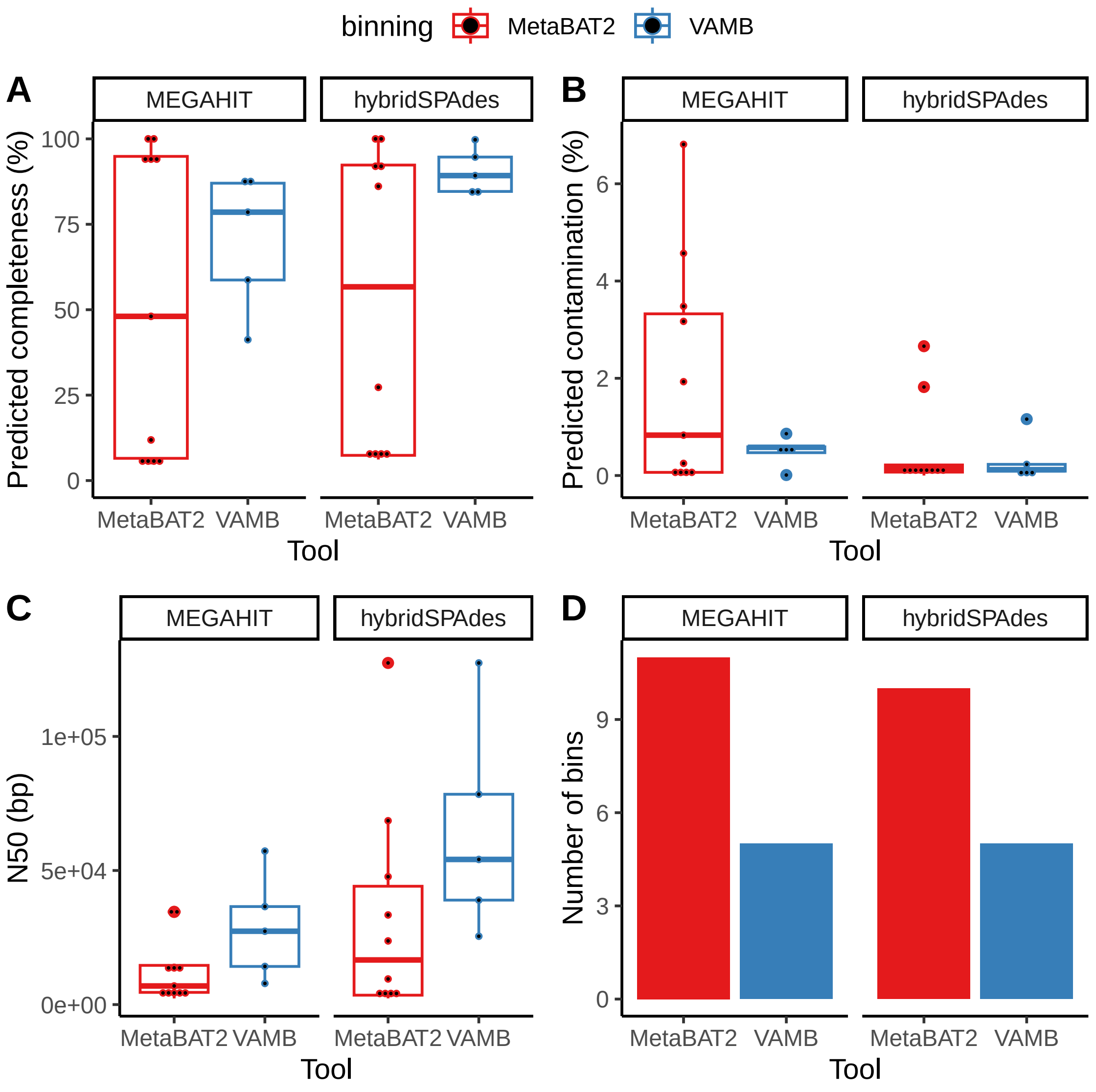

Figure S5: StrainMake facilitates straightforward benchmarking of metagenomic binners prior to refinement. Shown are binning results for sample 0 across different contig binning tools and assemblies prior to refinement. **A.** Predicted contamination estimated by CheckM2. **B.** Predicted completeness estimated by CheckM2. **C.** Bin N50 values. **D.** Number of bins produced.

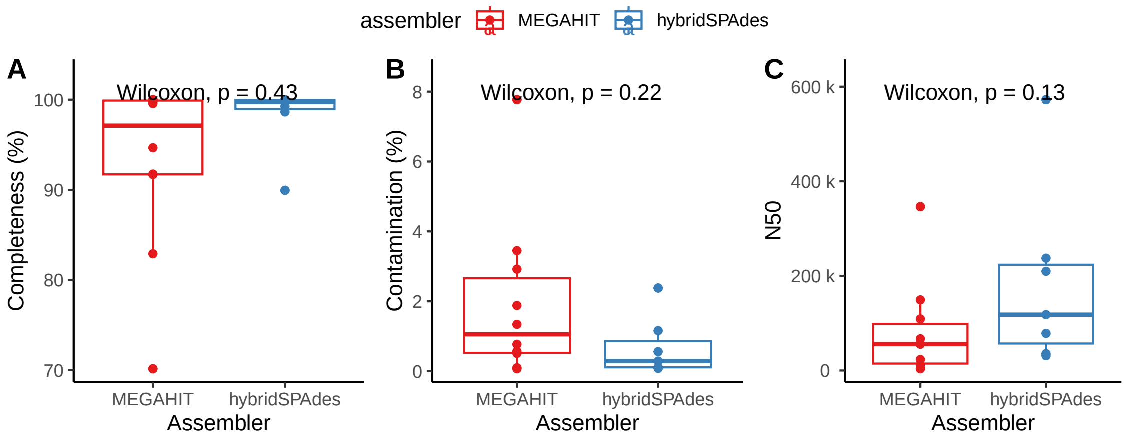

Figure S6: StrainMake enables benchmarking of refined bins across assemblers and facilitates the generation of high-quality MAGs. MAGs were generated from VAMB and MetaBAT2 outputs, refined with Binette, and dereplicated with dRep at 97% ANI across all samples. **A.** Predicted completeness estimated by CheckM2. **B.** Predicted contamination estimated by CheckM2. **C.** N50 values of refined bins.

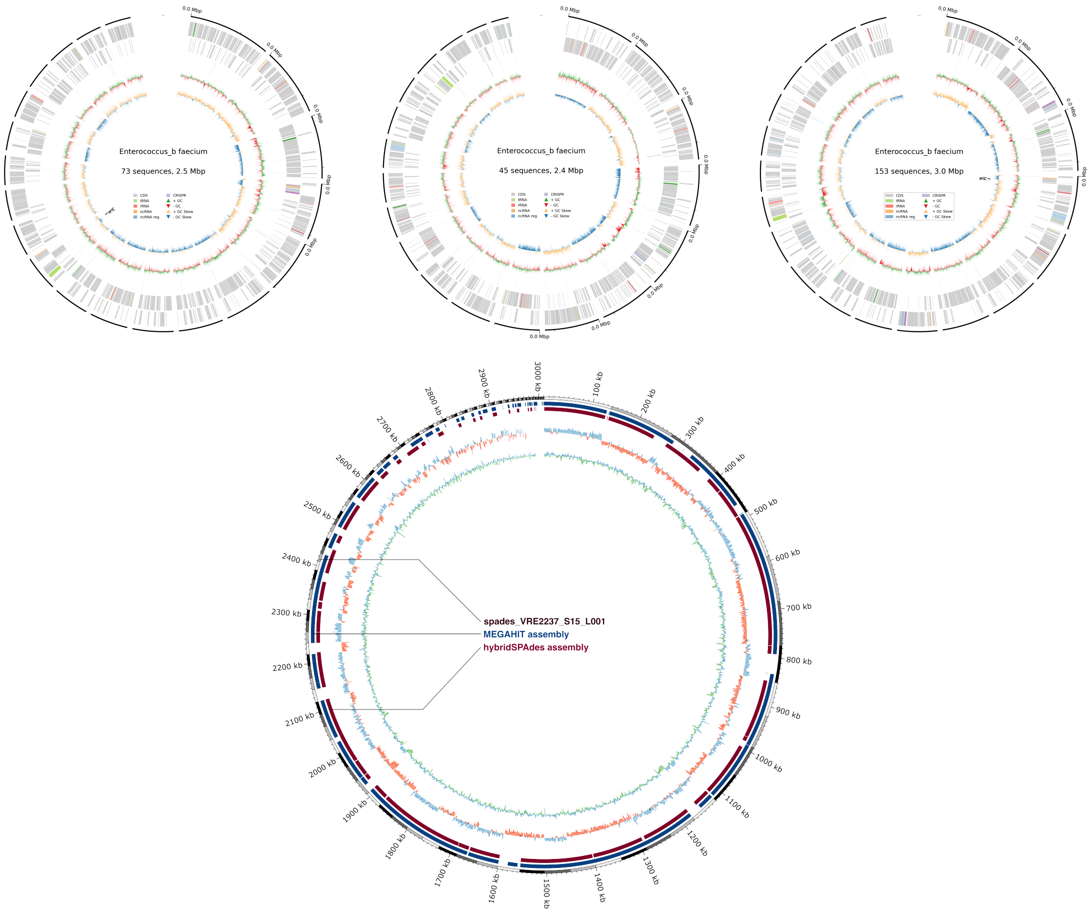

Figure S7: StrainMake facilitates the comparison of MAG annotations generated from different assembly strategies. **Top-left.** Bakta annotation of a MAG classified as *Enterococcus B faecium* obtained from MEGAHIT assemblies. **Top-centre.** Bakta annotation of a MAG classified as *E. faecium* obtained from hybridSPAdes assemblies. **Top-right.** For reference, Bakta annotation of one *E. faecium* strain genome used in the strain-madness simulation (spades_VRE2237_S15_L001). This example also illustrates that assemblies reconstructed with StrainMake are less fragmented than those produced by individual assemblers. **Bottom.** Whole-genome alignment of reconstructed *E. faecium* MAGs against one reference genome (spades_VRE2237_S15_L001), generated using mummer2circos (`https://github.com/metagenlab/mummer2circos`).

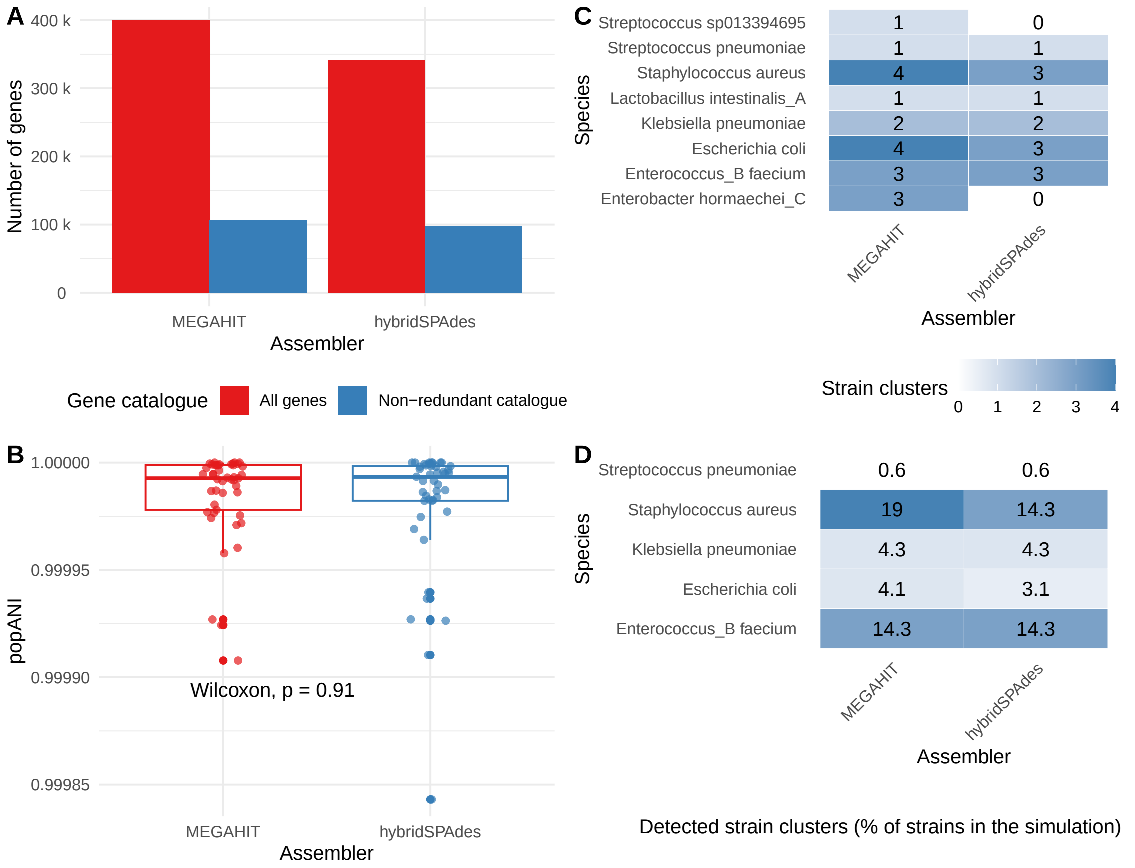

Figure S8: StrainMake enables both gene catalogue construction and strain-level comparative analyses. **A.** Comparison of gene catalogues generated by different assemblers. Red: genes predicted from assemblies across all samples; Blue: non-redundant gene catalogue obtained after pooling and dereplication. **B.** InStrain popANI values for *Enterococcus B faecium* across samples, stratified by assembler. **C.** Number of strain-level clusters, according to inStrain, per species genome reconstructed by StrainMake. **D.** Proportion of simulated strains detected as strain-level clusters by inStrain, expressed as a percentage: $\left(\frac{N_{\text{clusters}}}{N_{\text{simulated strains}}}\right) \times 100$. Higher values indicate closer agreement between the number of detected clusters and the number of simulated strains. Only recovered species with consistent taxonomic assignments between GTDB-Tk annotations of the MAGs and the CAMI II reference genomes were included.

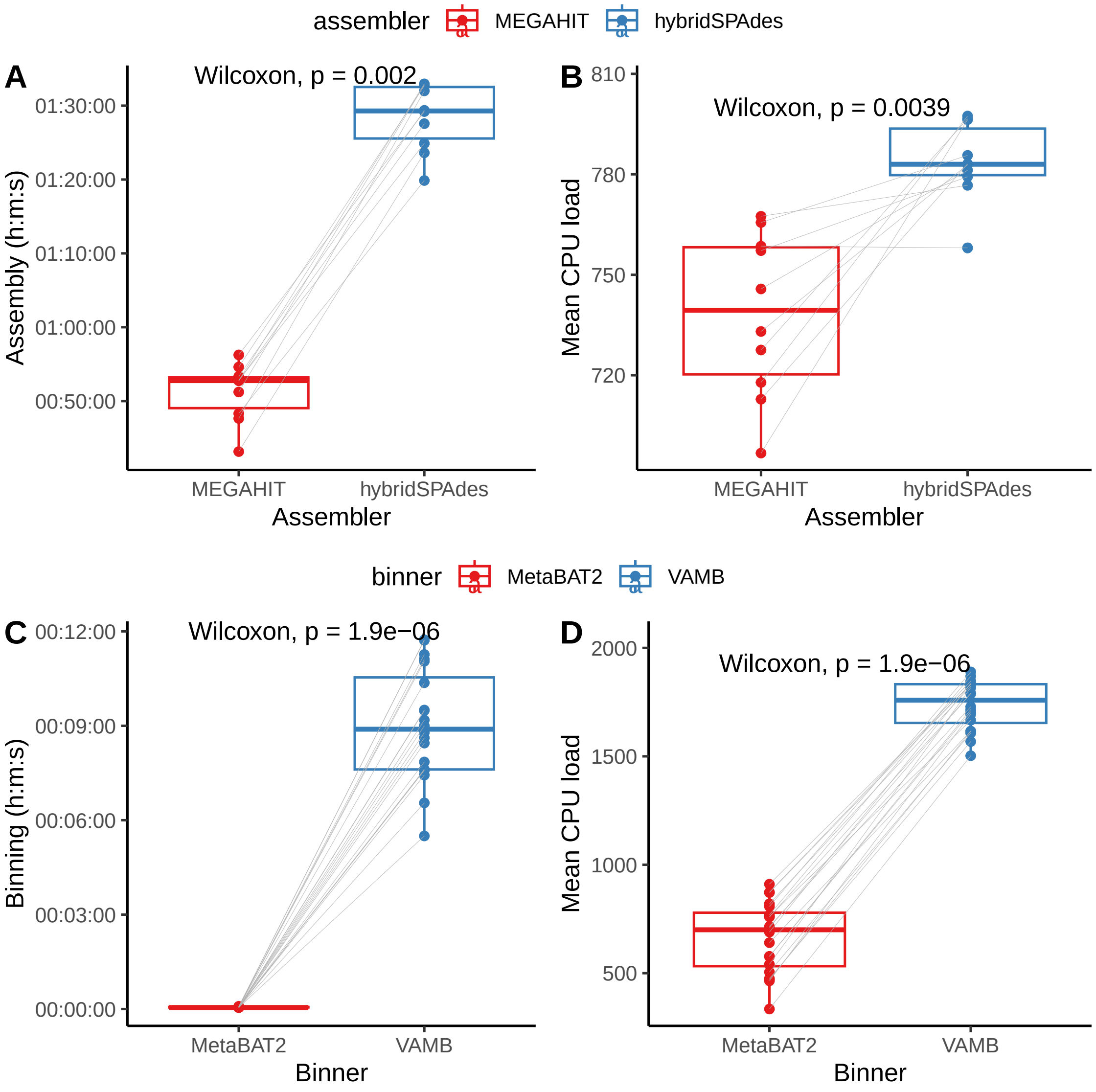

Figure S9: StrainMake facilitates benchmarking of computational performance across metagenomic tools. **A.** Comparison of assembly runtimes by assembler, aggregated across assemblies generated with different strategies. **B.** Comparison of mean CPU load during assembly by assembler. **C.** Comparison of binning runtimes by binner. **D.** Comparison of mean CPU load during binning by binner.

6 Analysis of CRC cohort PRJNA961076

Here we applied StrainMake to a real-life colorectal cancer (CRC) metagenomic cohort (PRJNA961076) consisting of Illumina MiSeq paired-end reads [Grion et al., 2024]. After preprocessing, samples had a mean sequencing depth of $2 \times 27{,}136{,}271 \pm 5{,}015{,}134$ paired-end reads per sample (Fig. S10). We additionally processed the cohort after downsampling reads to two target depths ($2 \times$ 5M and $2 \times$ 10M paired reads per sample) using Rasusa [Hall, 2022] to evaluate the effect of per-sample sequencing depth on assembly and MAG recovery. Downsampled FASTQs were run through the same StrainMake workflow and downstream analyses as the full-depth samples.

In this real CRC cohort, sequencing depth had clear effects on several layers of the StrainMake workflow. Assembly statistics were significantly affected by downsampling, indicating that reducing read depth alters contiguity and total reconstructed sequence length

(Fig. S11). The number of refined bins recovered per sample did not differ significantly between CRC and healthy individuals at any sequencing depth (Wilcoxon CRC vs Healthy: full-depth $p = 0.34$; 2×10M $p = 0.47$; 2×5M $p = 0.86$), indicating that host phenotype did not substantially affect bin recovery in this dataset (Fig. S12A). The number of recovered bins was positively correlated with species richness estimated from METEOR profiles, but this relationship was less pronounced at lower sequencing depth, consistent with reduced sensitivity of bin recovery when fewer reads are available (Fig. S12B).

At the MAG level, downsampling did not measurably affect completeness or contamination distributions, whereas MAG contiguity (N50) was significantly reduced, indicating that lower sequencing depth primarily increases fragmentation rather than degrading overall MAG quality (Fig. S12C–E). A consistent negative relationship between MAG completeness and contamination was observed at all depths, further supporting the stability of MAG quality across downsampling conditions (Fig. S12F). As expected, gene catalogue size decreased with downsampling, reflecting reduced assembly breadth and gene recovery (Fig. S13A). At the strain level, the two example species with the strongest phenotype-associated popANI differences showed broadly similar popANI distributions across sequencing depths, suggesting that major strain-level signals remain interpretable after moderate downsampling (Fig. S13B–C).

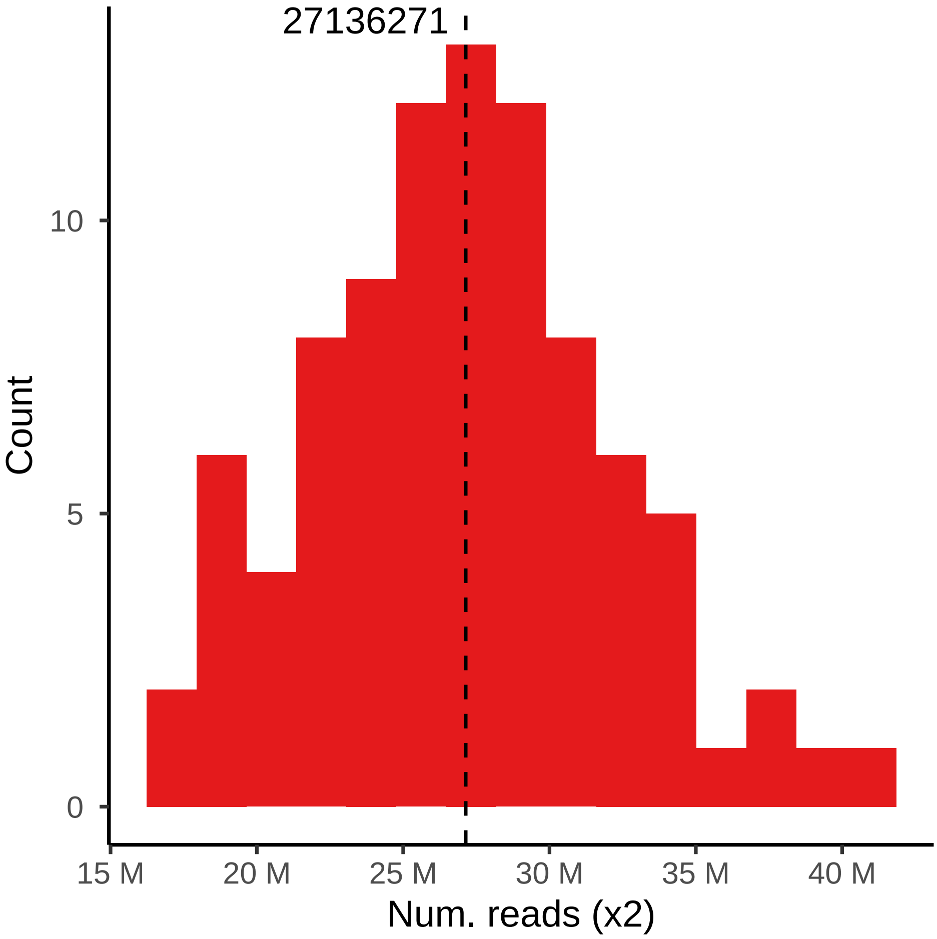

Figure S10: Sequencing depth of the PRJNA961076 cohort after StrainMake preprocessing. Per-sample paired-end read counts (after adapter trimming, quality filtering, and host decontamination); mean = $2 \times 27{,}136{,}271 \pm 5{,}015{,}134$ reads.

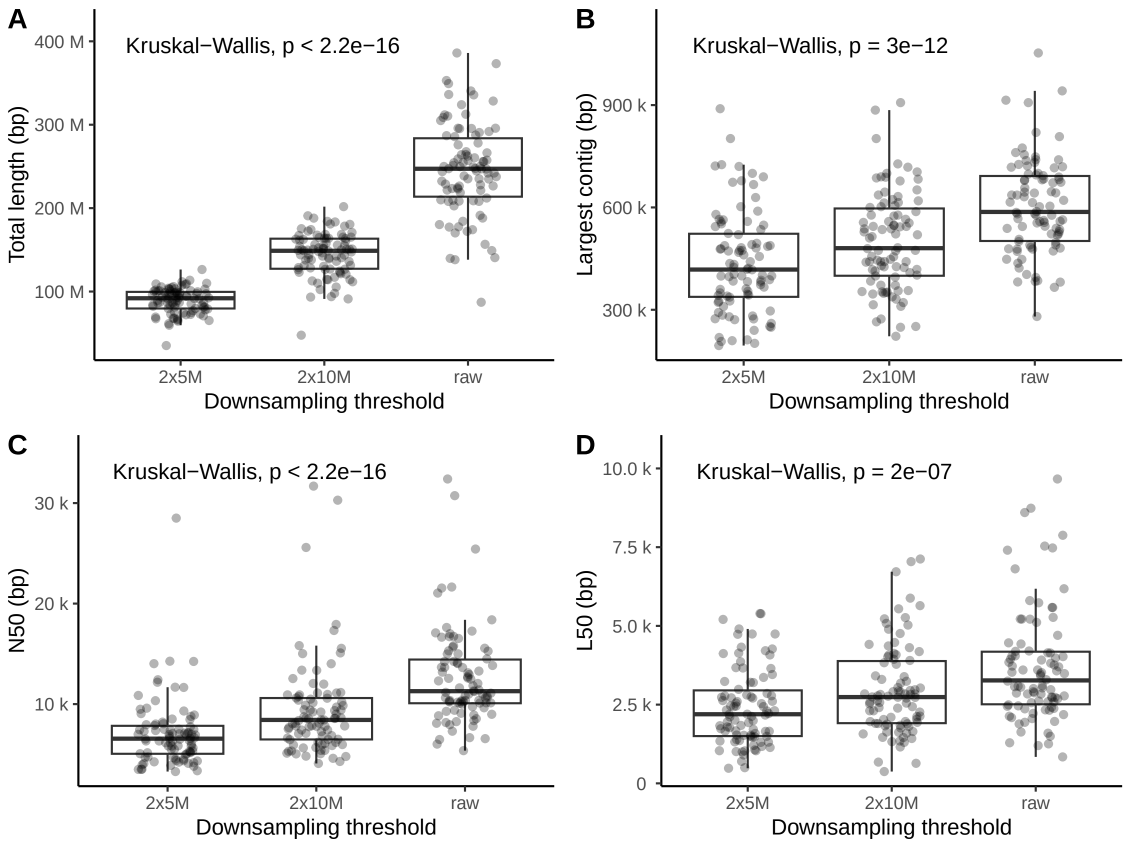

Figure S11: Distributions of assembly metrics for the PRJNA961076 cohort produced with MEGAHIT (short-read assemblies; $n = 90$). Boxplots are stratified by downsampling threshold (full-depth, 2×5M and 2×10M paired reads per sample). **A.** Total assembly length (bp; Kruskal–Wallis $p < 2.2 \times 10^{-16}$). **B.** Largest contig length (bp; Kruskal–Wallis $p = 3 \times 10^{-12}$). **C.** N50 (bp; Kruskal–Wallis $p < 2.2 \times 10^{-16}$). **D.** L50 (Kruskal–Wallis $p = 2 \times 10^{-7}$).

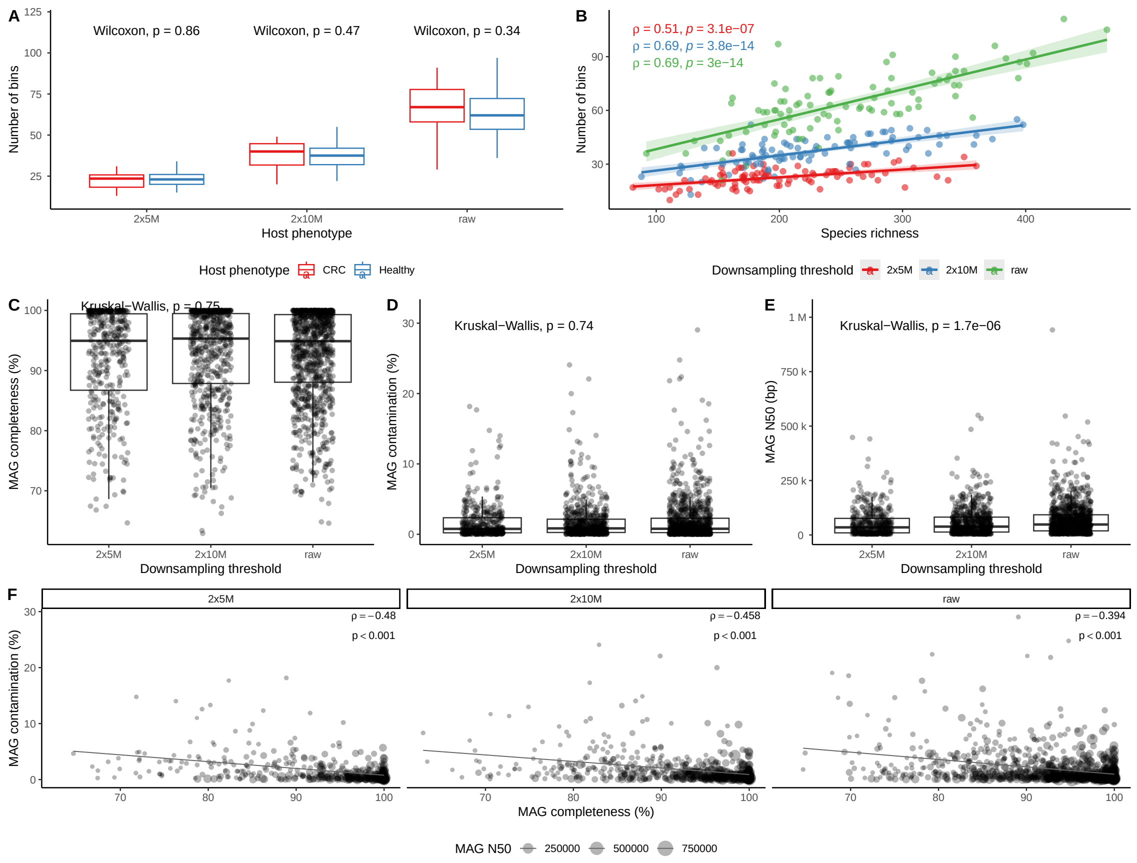

Figure S12: MAG-level summaries for the PRJNA961076 cohort. MAGs were generated from VAMB and MetaBAT2 outputs, refined with Binette, and dereplicated with dRep at 97% ANI across all samples. **A.** Number of refined bins recovered per sample after Binette refinement, stratified by host phenotype (CRC $n = 30$; Healthy $n = 60$). Wilcoxon (CRC vs Healthy) p-values by downsampling threshold: 2×5M, $p = 0.86$; 2×10M, $p = 0.47$; full-depth (raw), $p = 0.34$. **B.** Relationship between species richness (calculated with METEOR) and number of bins recovered per sample, stratified by downsampling; Spearman correlations — 2×5M: $\rho = 0.51$, $p = 3.1 \times 10^{-7}$; 2×10M: $\rho = 0.69$, $p = 3.8 \times 10^{-14}$; full-depth (raw): $\rho = 0.69$, $p = 3 \times 10^{-14}$. **C.** CheckM2-predicted completeness (%); Kruskal–Wallis $p = 0.75$. **D.** CheckM2-predicted contamination (%); Kruskal–Wallis $p = 0.74$. **E.** MAG contig N50 (bp); Kruskal–Wallis $p = 1.7 \times 10^{-6}$. **F.** Relationship between MAG completeness and contamination (faceted by downsampling); Spearman correlations — 2×5M: $\rho = -0.48$, $p < 0.001$; 2×10M: $\rho = -0.458$, $p < 0.001$; full-depth (raw): $\rho = -0.394$, $p < 0.001$. Points correspond to individual MAGs.

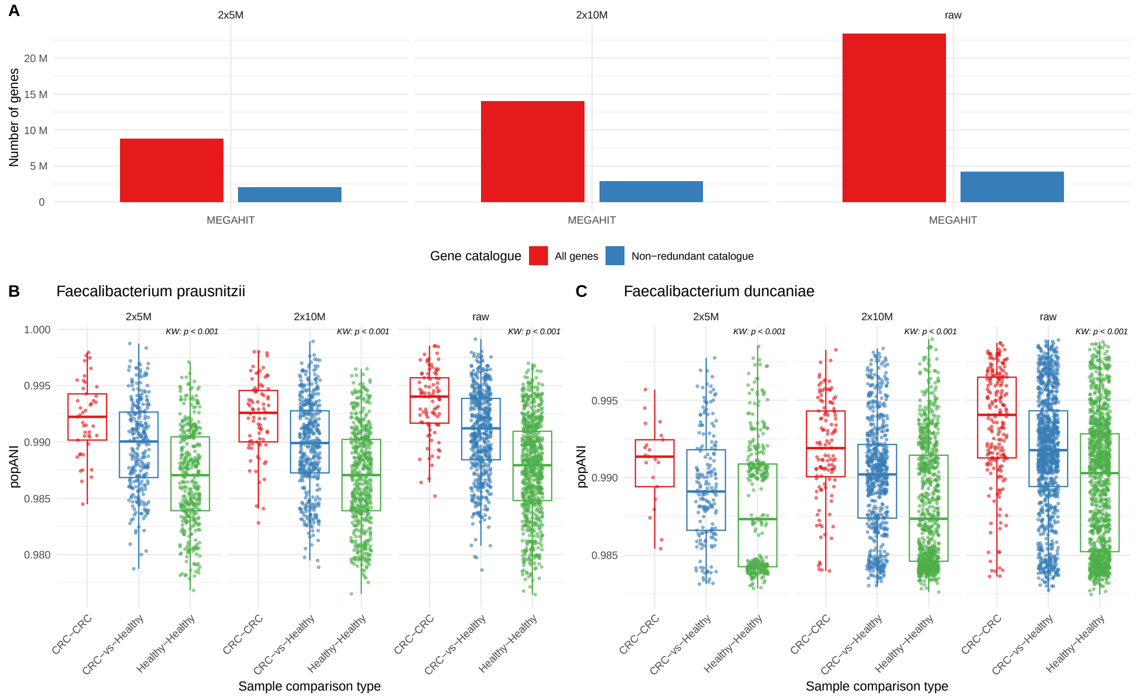

Figure S13: Gene catalogue construction and strain-level similarity for the PRJNA961076 cohort. **A.** Gene catalogue size by assembler and downsampling threshold. Bars show the total number of predicted genes for MEGAHIT (raw = 23,444,472; 2 × 10M = 13,993,787; 2 × 5M = 8,797,593) and the size of the non-redundant gene catalogue after pooling and clustering (raw = 4,212,574; 2 × 10M = 2,894,520; 2 × 5M = 2,036,396). **B–C.** Strain-level similarity across samples using inStrain genome-wide comparisons for the two species with the smallest FDR-adjusted Kruskal–Wallis p-values (*Faecalibacterium prausnitzii* and *Faecalibacterium duncaniae*). Comparisons are grouped as within-CRC (CRC–CRC), within-Healthy (Healthy–Healthy), or between phenotypes (CRC–Healthy). popANI represents the average nucleotide identity between compared populations; higher values indicate greater similarity.

7 Impact of sequencing coverage

To assess the impact of genome sequencing coverage on strain-level analysis, we performed a simulation study. We simulated metagenomes containing the following bacterial genomes under four coverage conditions, with five replicates per condition.

The four genome-coverage conditions are 2×, 10×, 20×, and 50×. We used MeSS [Chaabane et al., 2024] to simulate the samples with the Illumina option and the HiSeq 2500 error model.

The simulated reads were then processed with StrainMake to assemble reads, generate MAGs, perform taxonomic annotation, and run strain-level analyses.

Increasing sequencing coverage substantially improved both species recovery and the accuracy of strain-count estimation across the four conditions tested (2×–50×). Species recovery scaled with coverage; only three of five species were recovered at 2× coverage, increasing to all five at 10× coverage and above (Fig. S14). At the strain level, the accuracy of Floria's strain-count estimates improved with coverage, as reflected by a significant negative association between absolute estimation error and coverage (Spearman correlation: $\rho = -0.389$, $p = 6.31 \times 10^{-5}$) (Fig. S15). StrainMake's strain-level output improves with increasing sequencing coverage, and 10× coverage is sufficient for reliable recovery of the tested species and their estimated strain counts.

UHGG v2.0.2 identifier	Phylum	Species
MGYG000002388	Firmicutes	*Lacticaseibacillus paracasei*
MGYG000000045	Firmicutes	*Faecalibacillus intestinalis*
MGYG000103106	Firmicutes	*Faecalibacillus intestinalis*
MGYG000002422	Campylobacterota	*Campylobacter D jejuni*
MGYG000025950	Campylobacterota	*Campylobacter D jejuni*
MGYG000217975	Campylobacterota	*Campylobacter D jejuni*
MGYG000002469	Actinobacteriota	*Bifidobacterium breve*
MGYG000127504	Actinobacteriota	*Bifidobacterium breve*
MGYG000012994	Actinobacteriota	*Bifidobacterium breve*
MGYG000043917	Actinobacteriota	*Bifidobacterium breve*
MGYG000002506	Proteobacteria	*Escherichia coli D*
MGYG000061487	Proteobacteria	*Escherichia coli D*
MGYG000177156	Proteobacteria	*Escherichia coli D*
MGYG000173858	Proteobacteria	*Escherichia coli D*
MGYG000025790	Proteobacteria	*Escherichia coli D*

Table S2: Bacterial genomes used in the sequencing coverage simulation study.

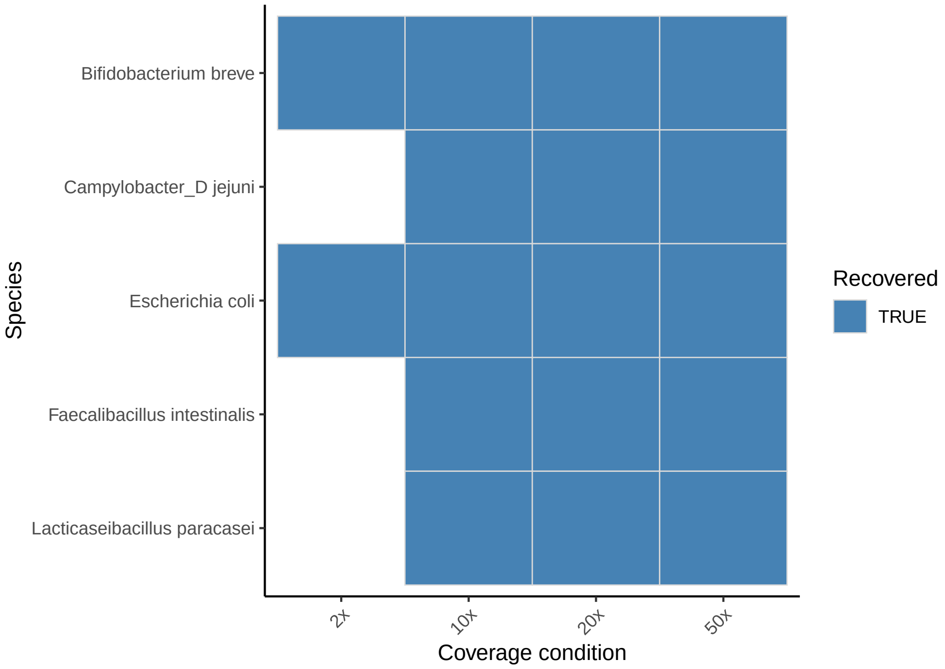

Figure S14: Recovery of introduced species across genome-coverage conditions. Heatmap derived from GTDB-Tk annotations of species-level MAGs reconstructed by StrainMake from MeSS-simulated metagenomes (five replicates per coverage condition). Each tile indicates whether the corresponding species was recovered (i.e., at least one MAG was assigned to that species) under the given sequencing-coverage condition (2×, 10×, 20×, 50×).

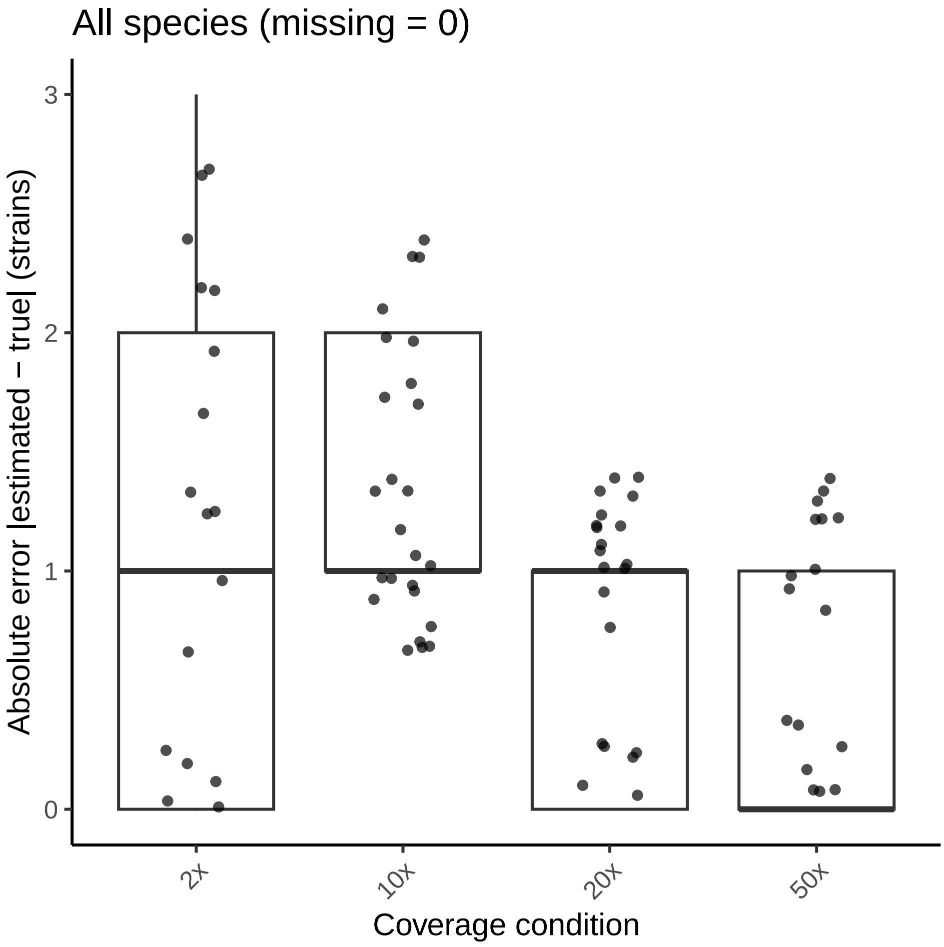

Figure S15: Effect of genome sequencing coverage on Floria strain-count estimation. As part of the StrainMake pipeline, Floria was run on species-level MAGs and reports an estimated strain count per contig; for each species in each replicate and coverage condition, we report the mean estimated strain count across contigs (rounded to the nearest integer). Estimation error was defined as the absolute deviation from the known strain count introduced in the simulation ($|\hat{s} - s|$). Species not recovered in a given replicate/condition (as assessed by GTDB-Tk; Fig. S14) were assigned an estimated strain count of 0. Absolute error showed a negative association with coverage (Spearman correlation: $\rho = -0.389$, $p = 6.31 \times 10^{-5}$).

8 Impact of cross-contamination

To evaluate the robustness of StrainMake to sample cross-contamination, we generated *in silico* contaminated read sets from real Illumina data (CRC cohort PRJNA961076). We randomly selected 16 paired-end samples from the cohort and partitioned them into eight pairs; within each pair, samples were used as mutual contamination sources.

We considered five contamination levels: 0%, 1%, 3%, 5%, and 10%. For a given target sample with n reads (per mate) and a target contamination level p, we sampled x reads (per mate) from its paired sample and appended them to the target FASTQs to produce a contaminated dataset, with x chosen such that $x/(n + x) = p$.

Read sampling was performed with seqkit [Shen et al., 2024] using the command `seqkit sample --number <x> --rand-seed <seed>`, and contaminated FASTQs were produced by concatenating the original target reads with the sampled contaminant reads.

In this controlled cross-contamination experiment (eight sample pairs; contamination

levels 0%, 1%, 3%, 5%, 10%), contamination produced measurable effects at multiple analytical scales but with important caveats. Assemblies were sensitive to added contaminant reads: Friedman tests indicated significant differences across contamination levels for N50, L50, and total assembly length (Fig. S16), whereas largest-contig length did not differ. By contrast, cohort-level MAG quality distributions (CheckM2 completeness, contamination, MAG N50) did not show significant global differences across contamination rates (Kruskal–Wallis tests; $p > 0.05$), indicating that binning, refinement, and MAG quality were robust under these contamination scenarios (Fig. S17).

At the strain level, per-species Kruskal–Wallis tests with Benjamini–Hochberg correction identified a subset of taxa with altered Floria estimated strain counts (98 of 294 species with BH-adjusted $q < 0.05$; Fig. S18A). Notably, the susceptibility of a taxon to contamination-induced changes in strain-count estimates was strongly associated with its baseline prevalence (Sylph [Shaw and Yu, 2025] profiling): low-prevalence taxa (1–4 of 16 baseline samples; 0–25%) were substantially more likely to yield significant results (49.6% significant) than taxa prevalent in 25–100% of baseline samples (16–24% significant), and this dependence was supported by a chi-square test of independence ($\chi^2 = 33.13$, df = 3, $p < 0.001$; Fig. S18B). By contrast, inStrain microdiversity comparisons were largely robust to contamination: among 174 species tested for popANI differences across contamination levels, only one species (*Faecalibacterium sp900758465*; $q = 0.00190$) showed a significant effect after BH correction (Fig. S18C).

Overall, contamination can affect assembly metrics and, for a minority of species, Floria's strain-count estimates (with effects concentrated at higher contamination levels), while MAG-level quality metrics remain broadly stable and inStrain popANI comparisons are largely robust.

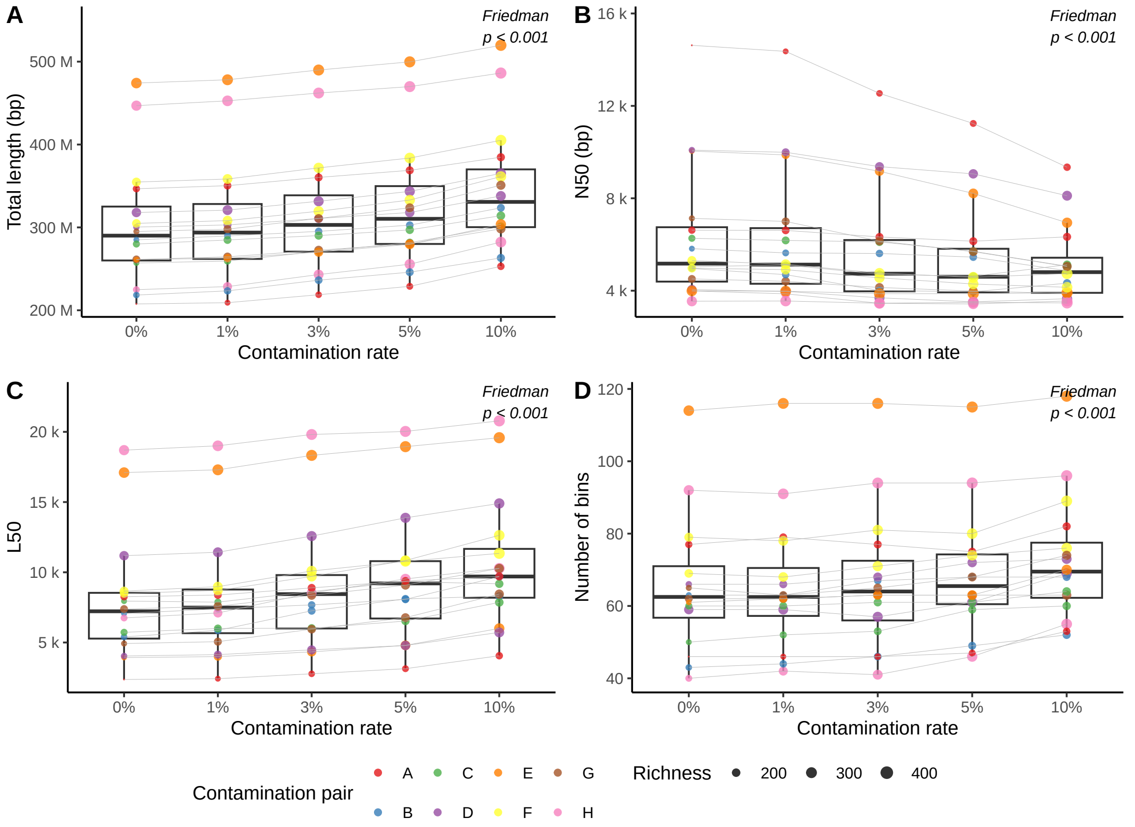

Figure S16: Effect of contamination rate on assembly metrics and number of recovered bins. Assemblies were generated for each sample across five contamination levels (0%, 1%, 3%, 5%, 10%). **A.** Total assembly length (bp). **B.** N50 (bp). **C.** L50. **D.** Number of refined bins recovered per sample after Binette refinement. Friedman tests: N50, $p = 6.43 \times 10^{-10}$; L50, $p = 8.65 \times 10^{-13}$; total assembly length, $p = 4.18 \times 10^{-13}$; number of refined bins, $p = 1.51 \times 10^{-9}$.

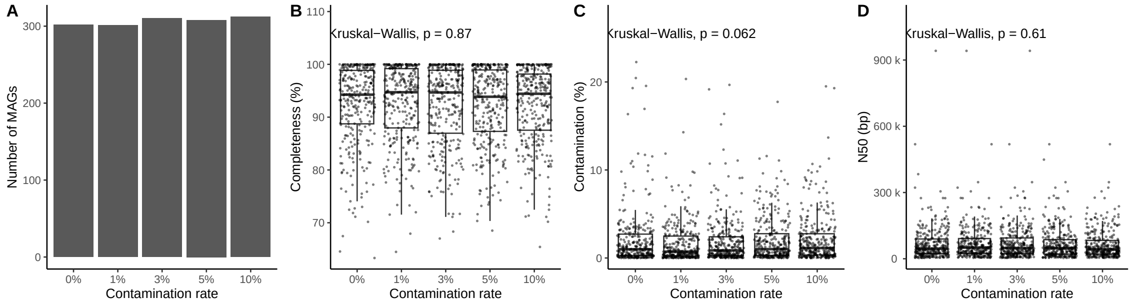

Figure S17: MAG quality metrics across contamination levels. MAGs were generated using StrainMake with VAMB and MetaBAT2, refined with Binette, pooled across samples, and dereplicated. **A.** Number of MAGs recovered per condition. **B.** Predicted completeness (%) estimated with CheckM2. **C.** Predicted contamination (%) estimated with CheckM2. **D.** MAG contig N50 (bp). Kruskal–Wallis tests: completeness, $p = 0.87$; contamination, $p = 0.062$; MAG N50, $p = 0.61$ (all not significant).

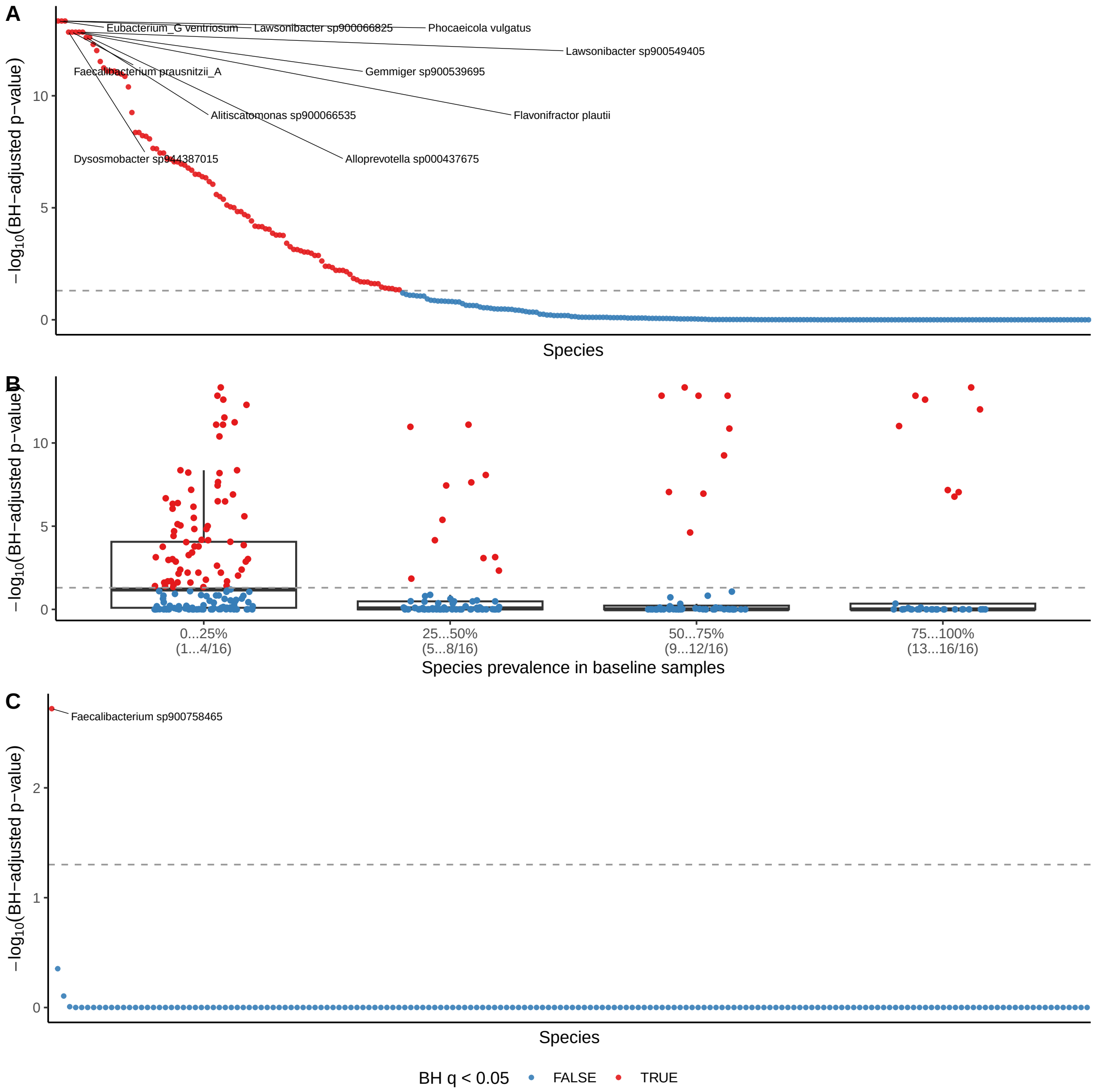

Figure S18: Effect of contamination rate on strain-level estimates and microdiversity metrics. **A.** BH-adjusted p-values (q-values) from Kruskal–Wallis tests comparing Floria estimated strain counts across five contamination levels for each species ($n = 294$ species tested; 98 significant after BH correction, $q < 0.05$). **B.** Association between species prevalence in baseline samples (derived from Sylph profiling) and Kruskal–Wallis significance. Low-prevalence species are more likely to show significant contamination effects (chi-square test: $\chi^2 = 33.13$, df $= 3$, $p < 0.001$). **C.** Robustness of inStrain microdiversity comparisons to contamination ($n = 174$ species); only one species showed a significant contamination effect.

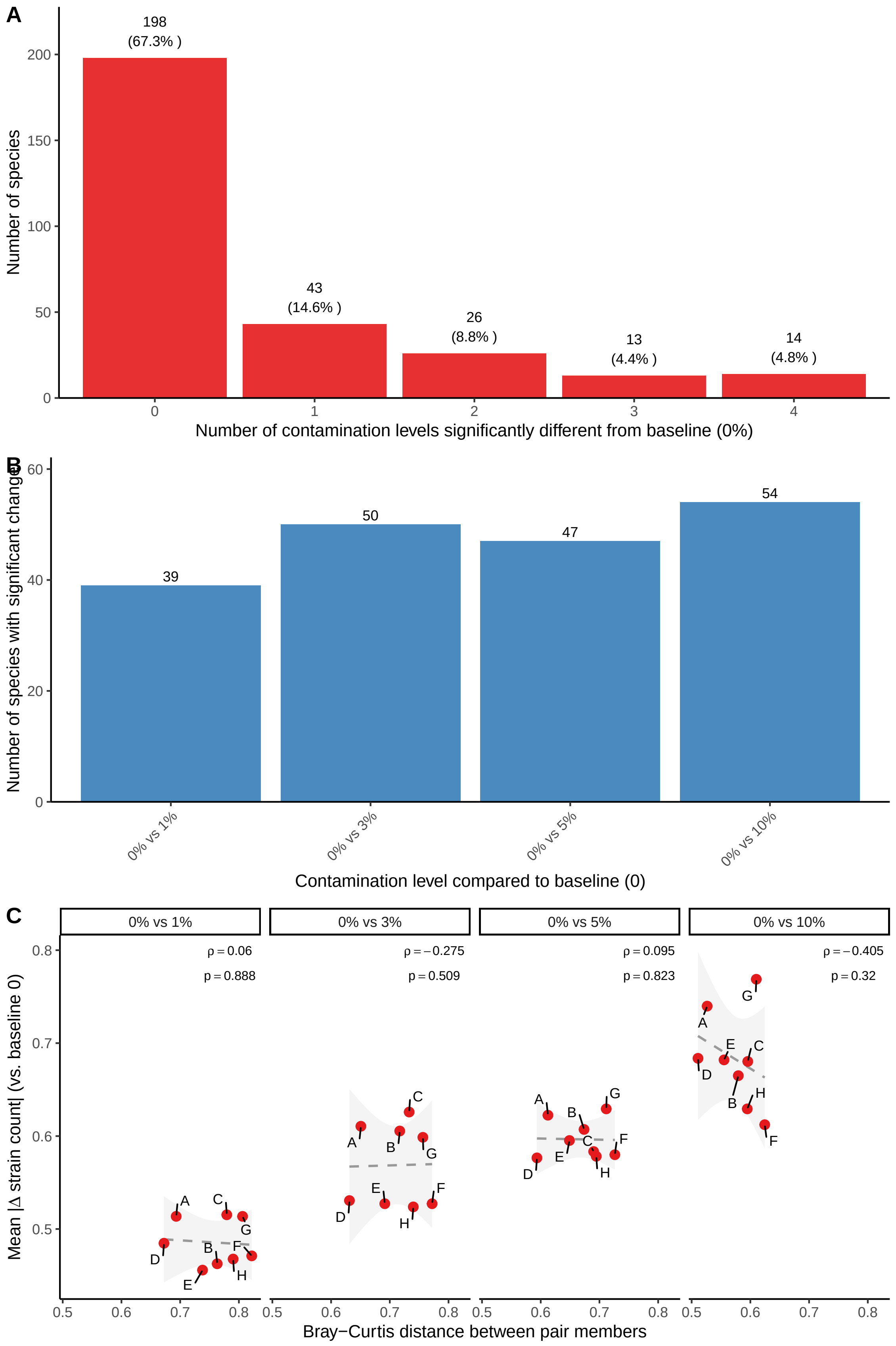

Figure S19: Pairwise Wilcoxon tests of Floria estimated strain counts across contamination scenarios. **A.** Number of species with at least one significant baseline comparison (BH-adjusted $q < 0.05$). **B.** Counts of species with significant change stratified by contamination pair. **C.** Relationship between pairwise Bray–Curtis dissimilarity of pair members and the mean absolute change in Floria's estimated strain counts per pair; Spearman correlations were not significant for any comparison.

References

S. Andrews. s-andrews/FastQC, 2025. original-date: 2017-12-21T11:48:51Z.

D. Antipov, A. Korobeynikov, J. S. McLean, and P. A. Pevzner. hybridSPAdes: an algorithm for hybrid assembly of short and long reads. *Bioinformatics*, 32(7):1009–1015, Apr. 2016. ISSN 1367-4811, 1367-4803. doi: 10.1093/bioinformatics/btv688.

A. Blanco-Míguez, F. Beghini, F. Cumbo, L. J. McIver, K. N. Thompson, M. Zolfo, P. Manghi, L. Dubois, K. D. Huang, A. M. Thomas, W. A. Nickols, G. Piccinno, E. Piperni, M. Punčochář, M. Valles-Colomer, A. Tett, F. Giordano, R. Davies, J. Wolf, S. E. Berry, T. D. Spector, E. A. Franzosa, E. Pasolli, F. Asnicar, C. Huttenhower, and N. Segata. Extending and improving metagenomic taxonomic profiling with uncharacterized species using MetaPhlAn 4. *Nature Biotechnology*, 41(11):1633–1644, Nov. 2023. ISSN 1087-0156, 1546-1696. doi: 10.1038/s41587-023-01688-w.

A. Cansdale and J. O. McInerney. MAGqual: a quality assessment tool for metagenome-assembled genomes. *Bioinformatics*, 40:btae523, 2024. doi: 10.1093/bioinformatics/btae523.

S. Chaabane, C. Hitte, P. Peterlongo, and R. Chikhi. MeSS: a tool for metagenomic sample simulation. *Bioinformatics*, 40:btae171, 2024. doi: 10.1093/bioinformatics/btae171.

P.-A. Chaumeil, A. J. Mussig, P. Hugenholtz, and D. H. Parks. GTDB-Tk v2: memory friendly classification with the genome taxonomy database. *Bioinformatics*, 38(23):5315–5316, Nov. 2022. ISSN 1367-4803, 1367-4811. doi: 10.1093/bioinformatics/btac672.

S. Chen. Ultrafast one-pass FASTQ data preprocessing, quality control, and deduplication using fastp. *iMeta*, 2(2):e107, May 2023. ISSN 2770-5986, 2770-596X. doi: 10.1002/imt2.107.

A. Chklovski, D. H. Parks, B. J. Woodcroft, and G. W. Tyson. CheckM2: a rapid, scalable and accurate tool for assessing microbial genome quality using machine learning. *Nature Methods*, 20(8):1203–1212, Aug. 2023. ISSN 1548-7091, 1548-7105. doi: 10.1038/s41592-023-01940-w.

A. Ghozlane, F. Thirion, F. P. Oñate, F. Gauthier, E. L. Chatelier, A. Annamalé, M. Almeida, S. Ehrlich, and N. Pons. Accurate profiling of microbial communities for shotgun metagenomic sequencing with Meteor2, Mar. 2025.

C. Grion et al. Gut microbiome signatures of colorectal cancer in southern brazil: exploring associations with clinical features and geographical and dietary factors. *Frontiers in Microbiology*, 14:1292490, 2024. doi: 10.3389/fmicb.2023.1292490.

M. B. Hall. Rasusa: Randomly subsample sequencing reads to a specified coverage. *Journal of Open Source Software*, 7:3941, 2022. doi: 10.21105/joss.03941.

H. Han, Z. Wang, and S. Zhu. Benchmarking metagenomic binning tools on real datasets across sequencing platforms and binning modes. *Nature Communications*, 16(1):2865, Mar. 2025. ISSN 2041-1723. doi: 10.1038/s41467-025-57957-6.

D. Hyatt, G.-L. Chen, P. F. LoCascio, M. L. Land, F. W. Larimer, and L. J. Hauser. Prodigal: prokaryotic gene recognition and translation initiation site identification. *BMC Bioinformatics*, 11(1):119, Dec. 2010. ISSN 1471-2105. doi: 10.1186/1471-2105-11-119.

D. D. Kang, F. Li, E. Kirton, A. Thomas, R. Egan, H. An, and Z. Wang. MetaBAT 2: an adaptive binning algorithm for robust and efficient genome reconstruction from metagenome assemblies. *PeerJ*, 7:e7359, July 2019. ISSN 2167-8359. doi: 10.7717/peerj.7359.

X. Kang, W. Zhang, Y. Li, X. Luo, and A. Schönhuth. HyLight: Strain aware assembly of low coverage metagenomes. *Nature Communications*, 15(1):8665, Oct. 2024. ISSN 2041-1723. doi: 10.1038/s41467-024-52907-0.

M. Kolmogorov, D. M. Bickhart, B. Behsaz, A. Gurevich, M. Rayko, S. B. Shin, K. Kuhn, J. Yuan, E. Polevikov, T. P. L. Smith, and P. A. Pevzner. metaFlye: scalable long-read metagenome assembly using repeat graphs. *Nature Methods*, 17(11):1103–1110, Nov. 2020. ISSN 1548-7091, 1548-7105. doi: 10.1038/s41592-020-00971-x.

B. Langmead and S. L. Salzberg. Fast gapped-read alignment with Bowtie 2. *Nature Methods*, 9(4):357–359, Apr. 2012. ISSN 1548-7091, 1548-7105. doi: 10.1038/nmeth.1923.

D. Li, C.-M. Liu, R. Luo, K. Sadakane, and T.-W. Lam. MEGAHIT: an ultra-fast single-node solution for large and complex metagenomics assembly via succinct *de Bruijn* graph. *Bioinformatics*, 31(10):1674–1676, May 2015. ISSN 1367-4811, 1367-4803. doi: 10.1093/bioinformatics/btv033.

H. Li. Minimap2: pairwise alignment for nucleotide sequences. *Bioinformatics*, 34(18): 3094–3100, Sept. 2018. ISSN 1367-4803, 1367-4811. doi: 10.1093/bioinformatics/bty191.

H. Liao, Y. Ji, and Y. Sun. High-resolution strain-level microbiome composition analysis from short reads. *Microbiome*, 11:183, 2023. doi: 10.1186/s40168-023-01615-w.

D. Machado, S. Andrejev, M. Tramontano, and K. R. Patil. Fast automated reconstruction of genome-scale metabolic models for microbial species and communities. *Nucleic Acids Research*, 46(15):7542–7553, Sept. 2018. ISSN 0305-1048, 1362-4962. doi: 10.1093/nar/gky537.

J. Mainguy and C. Hoede. Binette: a fast and accurate bin refinement tool to construct high quality Metagenome Assembled Genomes. *Journal of Open Source Software*, 9(102): 6782, Oct. 2024. ISSN 2475-9066. doi: 10.21105/joss.06782.

A. Mikheenko, V. Saveliev, and A. Gurevich. MetaQUAST: evaluation of metagenome assemblies. *Bioinformatics*, 32(7):1088–1090, Apr. 2016. ISSN 1367-4811, 1367-4803. doi: 10.1093/bioinformatics/btv697.

R. J. P. Newell, S. T. N. Aroney, J. Zaugg, P. Sternes, G. W. Tyson, and B. J. Woodcroft. Aviary: Hybrid assembly and genome recovery from metagenomes with Aviary, Mar. 2024.

J. N. Nissen, J. Johansen, R. L. Allesøe, C. K. Sønderby, J. J. A. Armenteros, C. H. Grønbech, L. J. Jensen, H. B. Nielsen, T. N. Petersen, O. Winther, and S. Rasmussen. Improved metagenome binning and assembly using deep variational autoencoders. *Nature Biotechnology*, 39(5):555–560, May 2021. ISSN 1087-0156, 1546-1696. doi: 10.1038/s41587-020-00777-4.

S. Nurk, D. Meleshko, A. Korobeynikov, and P. A. Pevzner. metaSPAdes: a new versatile metagenomic assembler. *Genome Research*, 27(5):824–834, May 2017. ISSN 1088-9051, 1549-5469. doi: 10.1101/gr.213959.116.

M. R. Olm, C. T. Brown, B. Brooks, and J. F. Banfield. dRep: a tool for fast and accurate genomic comparisons that enables improved genome recovery from metagenomes through de-replication. *The ISME Journal*, 11(12):2864–2868, Dec. 2017. ISSN 1751-7362, 1751-7370. doi: 10.1038/ismej.2017.126.

M. R. Olm, A. Crits-Christoph, K. Bouma-Gregson, B. A. Firek, M. J. Morowitz, and J. F. Banfield. inStrain profiles population microdiversity from metagenomic data and sensitively detects shared microbial strains. *Nature Biotechnology*, 39(6):727–736, June 2021. ISSN 1087-0156, 1546-1696. doi: 10.1038/s41587-020-00797-0.

S. Pan, X.-M. Zhao, and L. P. Coelho. SemiBin2: self-supervised contrastive learning leads to better MAGs for short- and long-read sequencing. *Bioinformatics*, 39(Supplement_1): i21–i29, June 2023. ISSN 1367-4803, 1367-4811. doi: 10.1093/bioinformatics/btad209.

D. H. Parks, M. Imelfort, C. T. Skennerton, P. Hugenholtz, and G. W. Tyson. CheckM: assessing the quality of microbial genomes recovered from isolates, single cells, and metagenomes. *Genome Research*, 25(7):1043–1055, July 2015. ISSN 1088-9051, 1549-5469. doi: 10.1101/gr.186072.114.

O. Schwengers, L. Jelonek, M. A. Dieckmann, S. Beyvers, J. Blom, and A. Goesmann. Bakta: rapid and standardized annotation of bacterial genomes via alignment-free sequence identification: Find out more about Bakta, the motivation, challenges and applications, here. *Microbial Genomics*, 7(11), Nov. 2021. ISSN 2057-5858. doi: 10.1099/mgen.0.000685.

J. Shaw and Y. W. Yu. Rapid and accurate metagenome and isolate sequencing with sylph, a seed-count k-mer method for species-level profiling and genome querying. *Nature Biotechnology*, 42:261–269, 2025. doi: 10.1038/s41587-024-02412-y.

J. Shaw, J.-S. Gounot, H. Chen, N. Nagarajan, and Y. W. Yu. Floria: fast and accurate strain haplotyping in metagenomes. *Bioinformatics*, 40(Supplement_1):i30–i38, June 2024. ISSN 1367-4803, 1367-4811. doi: 10.1093/bioinformatics/btae252.

W. Shen, B. Sipos, and L. Zhao. SeqKit2: A Swiss army knife for sequence and alignment processing. *iMeta*, 3(3):e191, June 2024. ISSN 2770-5986, 2770-596X. doi: 10.1002/imt2.191.

M. Steinegger and J. Söding. MMseqs2 enables sensitive protein sequence searching for the analysis of massive data sets. *Nature Biotechnology*, 35(11):1026–1028, Nov. 2017. ISSN 1546-1696. doi: 10.1038/nbt.3988.

J. Tamames and F. Puente-Sánchez. SqueezeMeta, A Highly Portable, Fully Automatic Metagenomic Analysis Pipeline. *Frontiers in Microbiology*, 9:3349, Jan. 2019. ISSN 1664-302X. doi: 10.3389/fmicb.2018.03349.

G. V. Uritskiy, J. DiRuggiero, and J. Taylor. MetaWRAP—a flexible pipeline for genome-resolved metagenomic data analysis. *Microbiome*, 6(1):158, Dec. 2018. ISSN 2049-2618. doi: 10.1186/s40168-018-0541-1.

A. J. van der Walt, M. W. van Goethem, and D. A. Cowan. Assembling metagenomes, one community at a time. *BMC Genomics*, 18:521, 2017. doi: 10.1186/s12864-017-3918-9.

T. Van Rossum, P. Ferretti, O. M. Maistrenko, and P. Bork. Diversity within species: interpreting strains in microbiomes. *Nature Reviews. Microbiology*, 18(9):491–506, Sept. 2020. ISSN 1740-1534. doi: 10.1038/s41579-020-0368-1.

J. Vollmers, S. Wiegand, and M. A.-K. Ferrer. Comparing and evaluating metagenome assembly tools from a microbiologist's perspective – not only size matters! *PLoS ONE*, 12:e0169662, 2017. doi: 10.1371/journal.pone.0169662.

J. Yepes-García and L. Falquet. 2Pipe starts with a question: matching you with the correct pipeline for MAG reconstruction. *mSystems*, 11(2):e00844–25, Feb. 2026. ISSN 2379-5077. doi: 10.1128/msystems.00844-25.
